# CLAME: a new alignment-based binning algorithm allows the genomic description of a novel Xanthomonadaceae from the Colombian Andes

**DOI:** 10.1186/s12864-018-5191-y

**Published:** 2018-12-11

**Authors:** Andres Benavides, Juan Pablo Isaza, Juan Pablo Niño-García, Juan Fernando Alzate, Felipe Cabarcas

**Affiliations:** 1 Grupo SISTEMIC, Ingeniería Electrónica, Facultad de Ingeniería, Universidad de Antioquia UdeA, Calle 70 No, 52-21 Medellín, Colombia; 2 Centro Nacional de Secuenciación Genómica-CNSG, Sede de Investigación Universitaria-SIU, Universidad de Antioquia UdeA, Calle 70 No, 52-21 Medellín, Colombia; 3 Escuela de Microbiología, Universidad de Antioquia UdeA, Calle 70 No, 52-21 Medellín, Colombia; 4 Grupo de Parasitología, Departamento de Microbiología y Parasitología, Facultad de Medicina, Universidad de Antioquia UdeA, Calle 70 No, 52-21 Medellín, Colombia

**Keywords:** Algorithm, Binning, Draft genome, Hot spring, Metagenomics, *Xanthomonadaceae*

## Abstract

**Background:**

Hot spring bacteria have unique biological adaptations to survive the extreme conditions of these environments; these bacteria produce thermostable enzymes that can be used in biotechnological and industrial applications. However, sequencing these bacteria is complex, since it is not possible to culture them. As an alternative, genome shotgun sequencing of whole microbial communities can be used. The problem is that the classification of sequences within a metagenomic dataset is very challenging particularly when they include unknown microorganisms since they lack genomic reference. We failed to recover a bacterium genome from a hot spring metagenome using the available software tools, so we develop a new tool that allowed us to recover most of this genome.

**Results:**

We present a proteobacteria draft genome reconstructed from a Colombian’s Andes hot spring metagenome. The genome seems to be from a new lineage within the family *Rhodanobacteraceae* of the class *Gammaproteobacteria*, closely related to the genus *Dokdonella*. We were able to generate this genome thanks to CLAME. CLAME, from Spanish “CLAsificador MEtagenomico”, is a tool to group reads in bins. We show that most reads from each bin belong to a single chromosome. CLAME is very effective recovering most of the reads belonging to the predominant species within a metagenome.

**Conclusions:**

We developed a tool that can be used to extract genomes (or parts of them) from a complex metagenome.

**Electronic supplementary material:**

The online version of this article (10.1186/s12864-018-5191-y) contains supplementary material, which is available to authorized users.

## Background

Bacterial populations have colonized almost every possible niche on Earth, including those considered harsh for most organisms. These extreme environments are those with a chemical composition or constraints imposed by the physical conditions where most organisms cannot survive. Thermophiles are present in several ecosystems where temperatures rise above 50 °C and reach up to 90 °C. They can grow optimally under these conditions [1], since they have the adaptations and the necessary enzymatic machinery to deal with the complications of living in these extreme environments. Therefore thermophiles are a potential source of thermostable proteins suitable for several industrial and biotechnological applications; then, the screening of novel thermophilic enzymes has become an important field of research. Although several thermostable enzymes have been recently described and characterized (e.g. [2–4]), thermophiles are still highly unexplored [5], especially because the majority of prokaryotic diversity cannot be cultured [6]. There have only been a few attempts to characterize enzymes or microorganisms from Neotropics hot springs (e.g. [7–11]) and just a handful of them (i.e. [10, 11]) used metagenomic approaches based on Next Generation Sequencing - NGS [12].

Since metagenomic NGS (from now on just metagenomic) approaches generate millions of short DNA reads of a few hundred bases [13], the challenge is to reconstruct the different species individual chromosomes from these reads. In a typical genomic experiment, most of the short reads belong to a single organism, and they can be assembled reliably using the tools that have been developed for this purpose (e.g. Newbler [14], Velvet [15], and Ray [16]). However, in a metagenomic experiment there is a mixture of reads from multiple species of a community [17]; moreover, the number of genomes and the abundance of reads from each species, in the sample, is unknown. These characteristics make the assembly process difficult, since there is a high risk of assembling reads from different organisms as a single chromosome. Tools like MetaVelvet [18], Ray Meta [19], MetAMOS [20], and SPAdes [21] use different approaches to address these issues and improve the assembly opportunities. However, these tools are far from perfect, and chimeric chromosomes can be assembled [22].

In order to reduce chimeric assemblies, researchers group reads in bins, based on their sequence similarity, to reduce the data complexity and to increase the likelihood of obtaining a reliable assembly. Tools like AMPHORA2 [17], MEGAN [23], MG-Rast [24], Kraken [25], Clark [26] or MetaBinG [27] use reference-based methods (i.e. supervised) that bin the reads or contigs into taxonomic clades based on pair-wise comparisons against reference databases, or pre-computed models. Similarly, there are reference-free methods (e.g. unsupervised) like MetaProb [28], BiMeta [29], MetaCluster [30], AbundanceBin [31] or CompostBin [32], that group reads using their genetic mutual similarities or their k-bases frequency composition, avoiding the pair-wise comparison step against reference databases. Supervised methods work fine in reconstructing genomes from well characterized or low-diversity communities, whose taxa have a good representation in reference databases; they exclude reads that come from less explored communities. In contrast, unsupervised methods are better when the species are poorly represented in databases, especially with long reads or contigs that increase the likelihood of finding genetic markers into a sequence to bin them correctly.

Although there are research publications that propose a draft genome of an unknown species extracted from a metagenome (eg [33, 34]), only few studies have reported the reconstruction of the complete genome of a thermophilic microbe (e.g. [35–37]). In these works, the process has been made mainly manual, using a combination of: Velvet [15], the study of the total coverage, k-mers characteristics and selecting contigs manually based on BLAST [38] results. In general, de-novo assembly of metagenome reads tends to generate short and chimeric contigs that are difficult to classify. Thus, the challenge of analyzing a metagenome is still open; we propose a tool that overcomes some of the limitation of traditional binning methods, mainly for metagenomes formed by unknown species.

Here, we introduce CLAME, a tool that groups metagenome reads in bins mainly from a single chromosome. The idea is to reduce the metagenomic complexity, to decreases the possibility of creating chimeric contigs and to improve the assembly speed. CLAME, from the Spanish “CLAsificador MEtagenomico”, is a C++ program that bins reads using a graph representation of the metagenome dataset. On the graph, reads are represented as nodes (vertices) and the overlap between two similar reads is represented as the edge that connects them. CLAME creates edges only on large exact matches between reads. This makes it very unlikely that two reads from different chromosome molecules can be clustered together. We found that this technique creates bins mostly from a single chromosome, while assigning most reads of one particular chromosome on a single bin. It is important to note that CLAME is not an assembly tool, it is a binning tool that groups reads as a preliminary step before genome assembly. We calibrated CLAME using public available NGS data from 454 and Illumina MiSeq platforms, and we tested it with a metagenomic dataset obtained from a never before studied Andean hot spring. CLAME allowed us to generate a high-quality draft genome (available in CLAME’s GitHub and on the NCBI’s project PRJNA431299) of a *Gammaproteobacteria* closely related to *Dokdonella* genus, which seems to represent a new lineage within the family *Rhodanobacteraceae*.

## Methods

CLAME groups metagenomic reads in bins using their biological and shotgun sequencing properties. The fundamental biological idea of CLAME is that exact matches, of a large number of bases, between reads is very unlikely if the reads do not come from same DNA chromosome. Furthermore, assuming that in a metagenome there is a genome sufficiently covered, and given that the sequencing errors is low (on platforms like Illumina Mi-seq or Roche’s 454), most reads from a DNA chromosome will have exact matches between them. This way CLAME reliably bins together most reads of each chromosome from a metagenome.

**Fig. 1:**
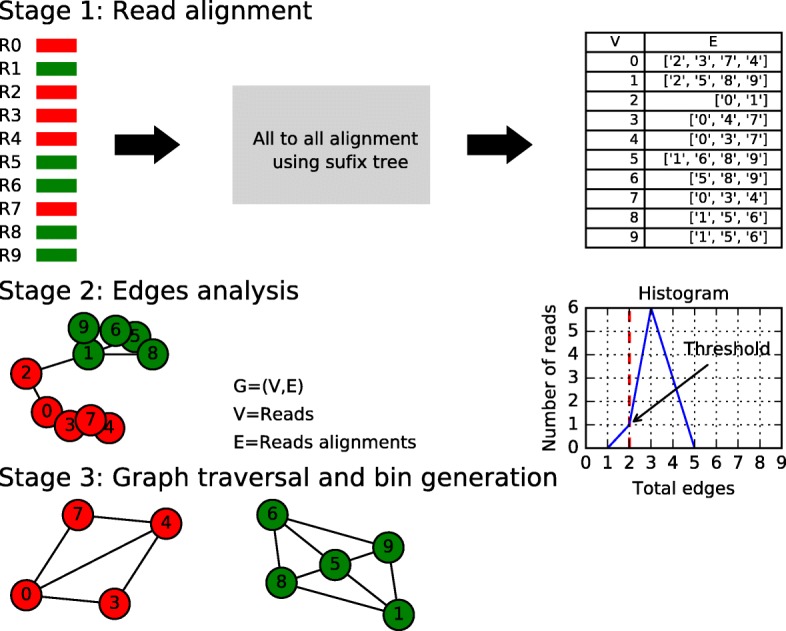
CLAME methodology. Stage 1) read alignment: the metagenome is composed by reads from different genomes (represented by the red and green colors); each read, represented by a single rectangle, is aligned against all the reads; an adjacency list shows all the alignments for each read. Stage 2) edges analysis: The graph representation indicates the relation of the reads; the reads that belong to a shared region can connect the subgroups (the green reads are connected to the green reads by the relation between read 1 and read 2); these connections usually make the number-of-edges histogram depart from a normal like form; then the histogram helps the user to set the number-of-edges thresholds on a range, in which a normal distribution is observed; It allows users to make bins with reads belonging to a normal-like connection profile. Stage 3) graph traversal and bin generation: the bins are generated by traveling the graph and reporting each subgraph (e.g. {1, 9, 6, 5, 8} green reads and {0, 3, 7, 4} red reads)

Initially, CLAME produces a graph with nodes (vertices) and edges, G = (V,E); while the reads are the nodes, the edges are the reads alignments. An edge between two reads is created only if they have an exact alignment of a large number of bases. Ideally, two reads from different DNA chromosomes will not align together, at least not in a considerable number of bases, and thus, the graph will represent the different organisms or chromosomes as organized subgraphs. The binning will thus follow naturally by traversing the graph, creating a bin for each connected subgraph. However, conserved regions, such as the ribosomal RNA genes, may generate edges between reads with different species memberships. CLAME considers the user-defined thresholds on the number of edges of a node when creating the bins. The user can define several thresholds to configure CLAME’s sensibility to the abundance of the species present which depends on the characteristics of the experiment. A detailed CLAME methodology is illustrated in Fig. 1 and explained in the next subsections.

### Read alignment stage

The read-overlap detection stage creates the edges of the graph. Algorithms like Needleman-Wush [39] and Smith-Waterman [40] were designed to find the optimal local alignment, the problem is that they have *O(n*^*2*^*)* computational times, where n is the number of bases of the reads. Thus, they are very slow for big datasets. To speed up alignment analysis, there are several algorithms that rely on a suffix/prefix tree representation of the dataset, such as suffix tree, enhanced suffix array or FM-index [41]. On these algorithms, all the reads are used to create a tree representation of them, and then, each read can be aligned to all others by searching it in the representation. In this case, the computational time can be reduced from *O(n*^*2*^*)* to *O(m + n)*, where m is the time to build the suffix tree, which is order n, and this way, the computational time can be reduced significantly.

CLAME uses a custom version of the suffix tree method: the Succinct Data Structure Library 2.0 [42]. With this library, we can find all the alignments of a query searching for a path in the tree. In the tree, descending from the root, each edge on the path matches a query. If there is a path for a query, it means that there is a substring and the reads in the path are the matches. To reduce computational time, CLAME only searches for exact alignments of *b* bases (forward and the reverse complement). The parameter “*b”* is the number-of-bases minimum-length alignment accepted, and it is set by the user. Using this information, CLAME creates the graph. It is represented as an adjacency list in which the first column represents the node and the second, the edges (the nodes that align in at least *b* bases). In an ideal case, the overlap stage must separate the graph, in sub-graphs, according to the number of chromosomes present in the metagenome. However, since there are sequencing errors and highly conserved genes, some reads can align in more than one species/chromosome, creating bins that include reads from more than one chromosome. To deal with this issue, CLAME uses edge analysis stage.

### Edge analysis stage

We have observed that the number of edges of a node is related to the abundance of that sequence on the metagenome. Furthermore, they follow a normal-like histogram. Using the adjacency list, generated in the read alignment stage, CLAME reports the reads’ number-of edges histogram of each bin. The number-of-edges histogram helps the user to set the thresholds, since a normal distribution is expected for the reads of a same chromosome, then the user can look at the graph and set the thresholds accordingly, to deal with the following problems. 1) nodes with a number of edges several times larger than the mean: Our experiments show that they are mainly produced by conserved zones of the DNA that are similar in several species. 2) nodes with a number of edges much smaller than the mean: we have observed that they are produced mainly by chimeric reads. Both of these problems make that reads from different DNA chromosomes end up being related.

Since the objective of CLAME is to create bins of reads from the single DNA chromosome, we allow the user to set thresholds on the number of edges. It allows users to eliminate reads with larger and smaller than the normal number of edges. CLAME takes users’ edge thresholds to redefine the graph and get connected subgraphs. The bins are generated by traveling the graph and reporting each subgraph.

### Graph traversal and bin generation

CLAME uses a greedy breadth-first search strategy to traverse the graph and to report each subgraph as a bin. It starts at an arbitrary node of a graph and explores the neighbor nodes first, before moving to the next neighbors’ level. It takes into consideration the edge thresholds to decide if the node is added to the bin or further analyzed. The process ends when no more reads can be added to the bin. At this point all the reads visited are reported as members of the same bin and a new seed is taken. This is done until all reads have been added to a bin. At the end, the bins and their reads are reported on output fasta files. CLAME allows the user to define a minimum bin size (number of reads) to avoid report singletons or very small bins.

### Simulated simple metagenome

A synthetic metagenome dataset was created using 289,917 reads of *Brucella canis* and 375,122 reads of *Mycobacterium tuberculosis,* both generated with the ROCHE’s 454 titanium platform and associated with the NCBI’s bioprojects PRJEB4803 and PRJEB8877, respectively. The reads were quality trimmed at Q30 using Prinseq [43]. The cleaned reads were concatenated on a simple multi-fasta file to get a total of 665,039 mixed reads that formed the Brucella-Mycobacterium synthetic metagenome. These reads were binned using CLAME, with at least 70 bases alignment. The parameters were determined experimentally, such that CLAME generated 2 bins for this metagenome (see Additional file 1 and Additional file 2 for the details).

**Fig. 2:**
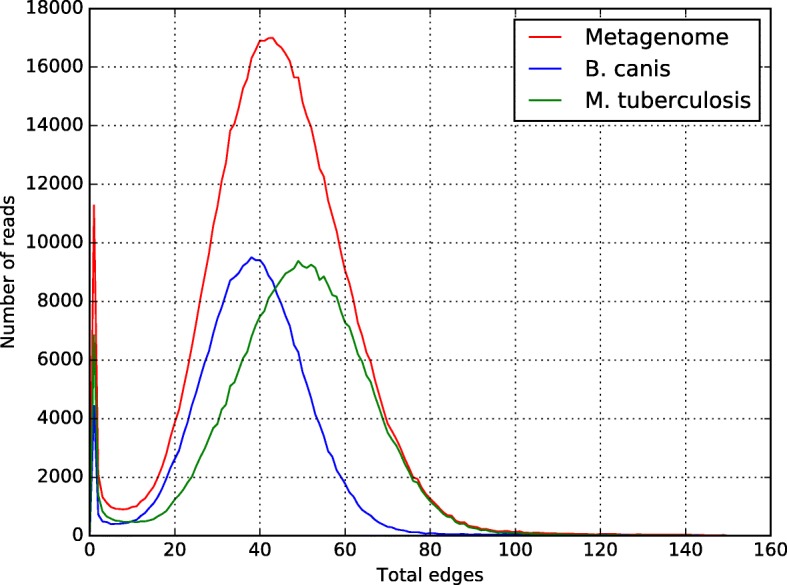
Number-of-edges histogram for the Brucella-Mycobacterium metagenome. The red line shows the metagenome histogram. The blue line shows the *B. canis*’ histogram and the green line shows the *M. tuberculosis’* histogram

*B. canis* and *M. tuberculosis* number of edges histogram is shown in Fig. 2, it was plotted with the in-house Python script plotHist.py; this script can be found as part of CLAME. Quality control for each bin was checked, by matching the content (read codes) of each bin against the original fastq files.

We also used MetaBinG [27], MetaProb [28], BiMeta [29], and AbundanceBin [31] tools to bin the metagenome. For the tools in which the number of bins or species can be specified, this parameter was set up to 2. Quality control for each tool was checked, by matching the content (read codes) of each bin against the original raw files. Table 1 shows the results of all the binning tools.

**Table 1:** Bins reported by each tool on the simulated metagenome. It also shows the number of reads that belong to each genome for each bin, and the time it took each tool to create the bins

Tool	Bins	Total reads by bin	B. Cannis	M. Tuberculosis	Time(m)
CLAME	2	353,876	0	353,876	8
		280,014	280,014	0	
BiMeta	2	8990	8683	307	49
		656,049	366,439	289,610	
MetaProb	2	368,642	2901	365,787	12
		296,397	287,062	9335	
AbundanceBin	2	659,892	288,233	371,659	85
		5142	1684	3458	
MetaBinG	2	600,615	5215	295,400	97
		338,650	267,794	70,856	

### Simulated multi-species metagenome

We created a metagenomic dataset based on the bacterial genomes of five species which were downloaded from the NCBI database: *Synechocystis,* SRA code DRR106442, *Dokdonella*, SRA code SRR4217676, *Hymnobacter*, SRA code SRR1334914, *Microbacteria*, SRA code SRR5493999 and *Rhizobium*, SRA code SRR5165471. For each species, the raw reads downloaded were merged into an extended single multifasta file using the Flash tool [44] (minimal identity parameter of 65 bases). In order to simulate different abundance levels, similar to the real spring-water metagenome, different amounts of extended reads were randomly taken from each dataset. Table 2 shows: the number of raw reads, the taxonomy of each species, the number of reads used (after using Flash to join read pairs), the size of the genome reported and the depth of each genome used. The final dataset was produced by concatenating the selected sequences into a single multifasta file.

**Table 2:** Species and total reads used to create the simulated multi-species metagenome. It shows the size of the original database, in reads and bases, the reads and bases used to create the metagenome, the size of the reported genome, and the depth calculated as the bases used divided by the genome size

Species	NCBI reference	Phylum/Class	Total reads	Total bases (Mbp)	Used reads	Used bases (Mpb)	Genome size (Mpb)	Depth(x)
Synechocystis	DRR106442	Cyanobacteria/Cyanobacteria	589,689	21.9	112,805	41.5	3.5	11.7
Dokdonella	SRR4217676	Proteobacteria/Gammaproteo-bacteria	376,022	80.5	376,022	80.5	4.6	17.41
Hymnobacter	SRR1334914	Bacteroidetes/ Cytophagia	2,917,298	958.5	37,599	12.3	5.0	2.4
Microbacteriaceae	SRR5493999	Actinobacteria/Actinobacteria	1,815,433	382.4	37,599	7.9	3.2	2.4
Rhizobium	SRR5165471	Proteobacteria/Alphaproteo-bacteria	1,152,754	242.2	37,599	7.9	4.5	1.7

**Fig. 3:**
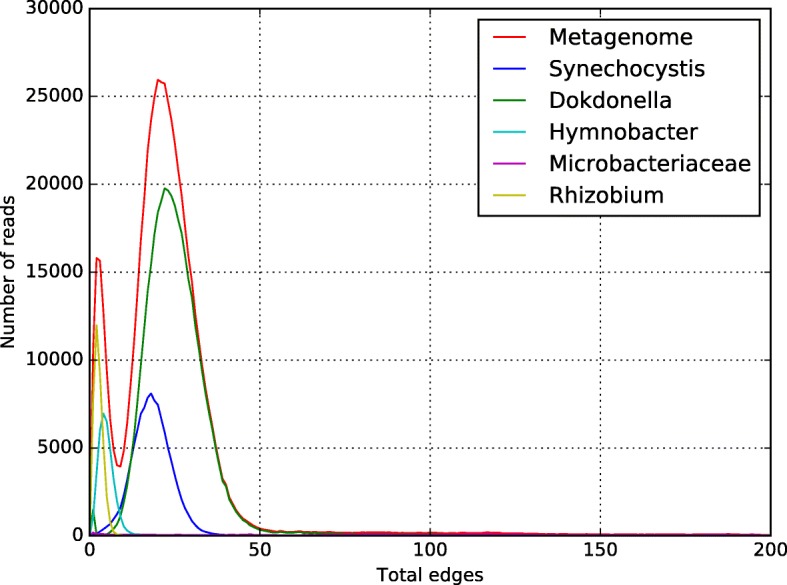
Number-of-edges histogram for the simulated multi-species metagenome. The red line shows the metagenome histogram. For each species an alone histogram is shown in different color lines

CLAME was executed using 70 bases alignment and no edge thresholds. The number of edges histogram is shown in Fig. 3 (generated with the script plotHist.py). Using the histogram CLAME was executed again using 70 bases and edge thresholds for the range 1, 51, 10,000. Quality control for each bin was manually checked, by matching the bins content versus the read codes from the original raw files (see Additional file 1 for the details).

We also executed MetaBinG [27], MetaProb [28], BiMeta [29], and AbundanceBin [31] tools with this metagenome. For the tools in which the number of bins or species can be specified, this parameter was configured to 5. Quality control for each tool was again checked, by matching the content of each bin against the original raw file codes. Table 3 compares these results versus CLAME’s results.

**Table 3:** Bins reported by the binning tools on the simulated multi-species metagenome. It also shows the number of reads that belong to each genome for each bin, and the time it took each tool to create the bins

Tool	Bins	Total reads by bin	Synechocystis	Dokdonella	Hymnobacter	Microbacteriaceae	Rhizobium	Time (m)
CLAME	7	21,182	21,182	0	0	0	0	3
		18,054	18,054	0	0	0	0	
		209,642	0	209,642	0	0	0	
		12,152	0	12,152	0	0	0	
		13,927	0	13,927	0	0	0	
		10,405	0	10,405	0	0	0	
		24,315	0	0	0	24,315	0	
BiMeta	1	601,624	112,805	376,022	37,599	37,599	37,599	32
MetaProb	5	361,966	1	341,866	108	7236	12,755	11
		27,977	508	12,139	1707	214	13,409	
		113,349	111,889	695	641	6	118	
		38,400	294	729	34,383	2446	548	
		59,932	113	20,593	760	27,697	10,769	
MetaBinG	5	410,033	30,727	302,805	23,480	19,944	33,081	120
		73,263	799	57,637	3915	9490	1423	
		61,401	56,764	2344	772	1211	310	
		24,966	18,955	3042	1079	870	1021	
		10,826	12	3800	6444	436	134	

### Illumina MiSeq metagenomic read set

This dataset corresponds to a real metagenomic sequencing experiment of human intestinal microbiota after a separation stage, where the intestinal protozoa *Cryptosporidium hominis* was enriched [45]. The original pair-ended reads cover the whole genome of this protozoan parasite, which is contained in 8 chromosomes. The reported reads belonging to *C. hominis* (1,066,460) were downloaded from SRA database Accession ERX1047563. The metagenome raw reads (9,052,596) (available in CLAME’s GitHub) were trimmed, using a minimum quality cutoff of Q30 using Prinseq [43] tool. Then the reads were merged into an extended single multifasta file using the Flash [44] tool. There were 6,052,596 left after these steps.

**Fig. 4:**
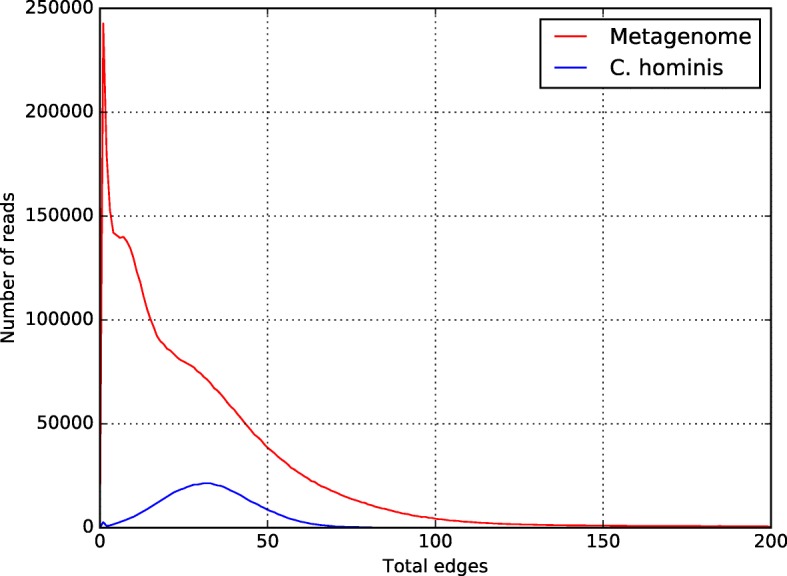
Number-of-edges histogram for the Illumina metagenome. The red line shows the metagenome histogram. The blue line shows the *C. hominis*’ histogram

The 6,052,596 reads were binned using CLAME with 100 bases alignment and custom edge thresholds. The distribution of the number of edges on the metagenome and the *C. hominis*’ read contribution was plotted using the python script plotHist.py (Fig. 4). We manually selected the bins that included reads from *C. hominis* genome (see Additional file 1 for the details).

**Fig. 5:**
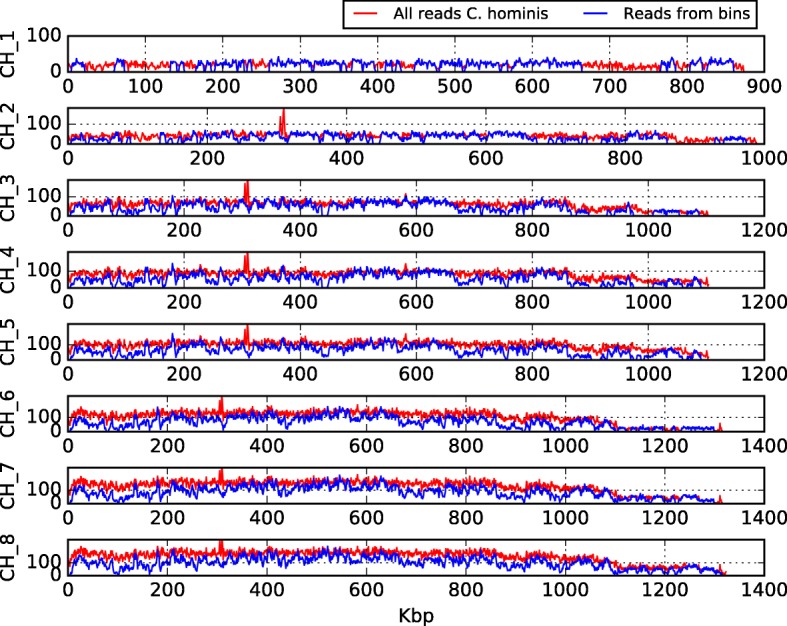
*C. hominis* whole genome coverage of the reads. The red line shows the coverage produced by the 728,463 original reads of the metagenome on each chromosome of the *C. hominis* genome. The blue line is the coverage of the 497,328 reads extracted from the selected bins with more than 500 reads

CLAME performance was measured using as a control the *C. hominis* genome reference (SRA Accession ERX1047563) by matching the coverage generated by the original reads versus the coverage generated by the binned reads. Bowtie2 [46] was used to map the reads to the reference. Figure 5 shows the obtained coverage; the data were plotted on the same figure using another in-house script plot (plotMapping.py).

Additionally we analyzed the biggest bins produced by CLAME (Tables 4 and 5). Each bin was assembled using Newbler [14], it was set to minimum identity (mi = 95) and minimum length (ml = 60). Annotation, for the Large contigs (> 500 bases) was done using AMPHORA2 [17], MEGAN [23] and RAIphy [47]. AMPHORA2 and RAIphy were executed with default parameters. For MEGAN, we generated a BLASTn-comparison file of the Large Contigs (> 500 bases) against a local NT (downloaded on May 2017) in XML format (see Additional file 1 for the details).

**Table 4:** Assembly statistics of the biggest bins reported by CLAME on the Illumina metagenome

Bin number	Total reads	Large contigs	Expected genome size (Mbp)	AVG contig length (bp)	Largest contig (bp)	N50	GC (%)
12	932,332	3211	6.0	1867	60,200	2639	37.67
9	514,053	447	3.6	8112	85,325	22,568	56.58

**Table 5:** Annotation of Newbler’s Large contigs assembled from the biggest bins reported by CLAME on the Illumina metagenome

Contigs	MEGAN		RAIphy		AMPHORA2	
	Total Contigs/Phylum	Total Contigs/Species	Total Contigs/Phylum	Total Contigs/Species	Total Contigs/Phylum	Total Contigs/Species
3211 from the bin 12	2856/ Firmicutes	2409/ Veillonella	2896/ Firmicutes	2437/ Veillonella	39/ Firmicutes	38/ Veillonella
447 from the bin 9	301/Actinobacteria	300 /Bifidobacterium	259 /Actinobacteria	237 /Bifidobacterium	40 /Actinobacteria	39/Bifidobacterium

### San Vicente hot spring metagenome

**Fig. 6:**
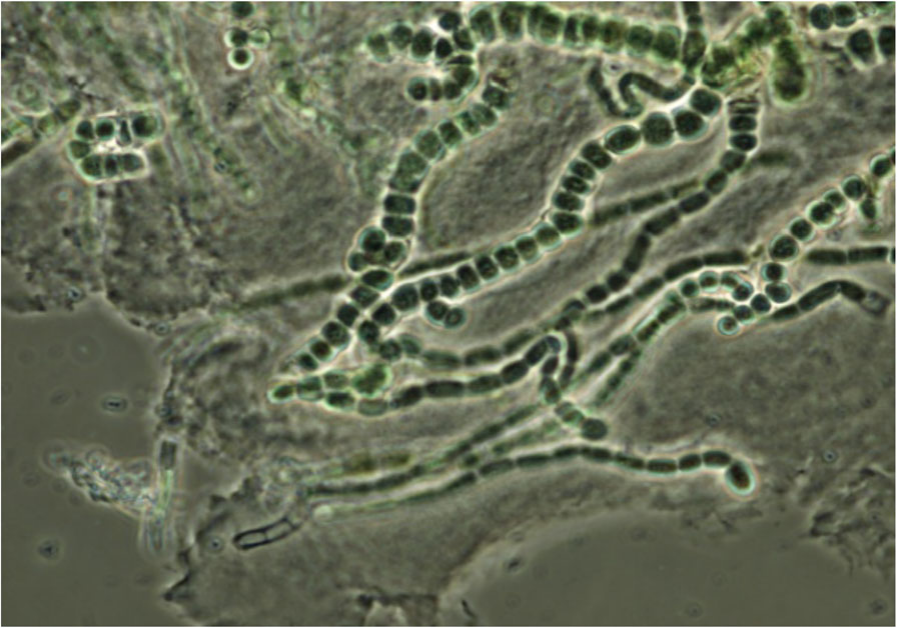
Microscopic photograph of Cyanobacteria growth culture from San Vicente water. A filamentous Cyanobacterium dominated the community and several small cells suggest that the desired reduction in the complexity of the community was achieved

San Vicente is a hot spring within the Cerro-Machin-Cerro-Bravo volcanic complex in Colombian Andes, located at 4° 50.25’ N and 75° 32.35’ W at an altitude of 1715 masl. It is characterized by waters with discharge temperatures above 60 °C (max. 91 °C), pH of 6.7 and high concentrations of chlorides. To reduce the complexity of the community, we incubated a sample of the hot spring (discharge temperature 64 °C) in a non-selective mineral medium, maintained at 45 °C with white light during 15 days (Fig. 6). We extracted the community DNA using PowerMax® Soil DNA Isolation Kit supplied by MOBIO Corporation [48], following the instructions of the manufacturer. The sample was sequenced using ROCHE’s 454 Titanium technology in 3/4 PTP at the Centro Nacional de Secuenciación Genómica - CNSG, Universidad de Antioquia, Medellin, Colombia. A total of 926,130 reads (available in CLAME’s GitHub and on the NCBI’s project PRJNA431299) were generated with a 300 bp average length. Raw reads were trimmed using Prinseq [43] tool to keep reads at least 50 bases long, and that at the 3′ the quality is at least 30 (see Additional file 1 for the details). Finally, a total of 900,370 quality reads were obtained for further processing steps. The analysis followed in two directions: 1) A de-novo metagenome assembly of the cleaned reads using popular state of the art tools (see below) and further comparison and annotation; 2) the binning of the quality reads using CLAME and further assembly and annotation using the biggest bin.

**Fig. 7:**
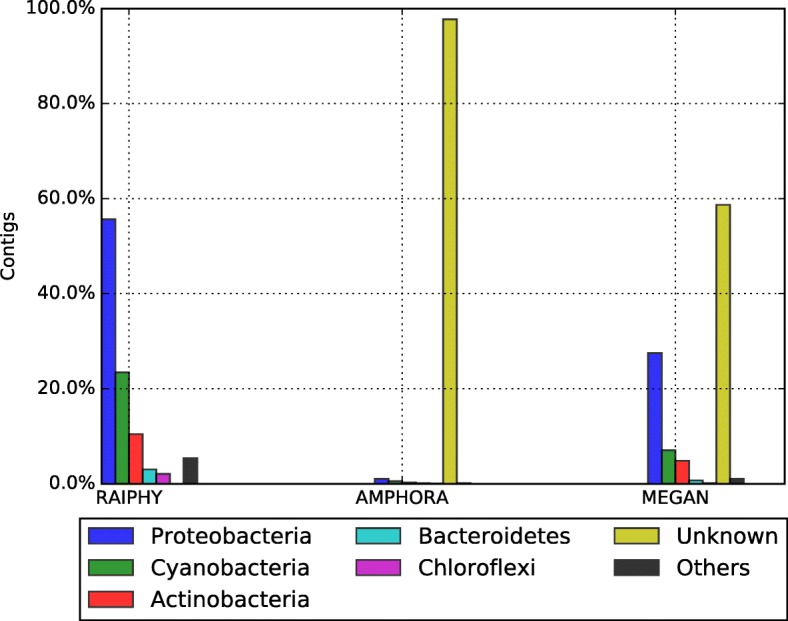
Phyla frequency reported by MEGAN, AMPHORA2 and RAIphy for the assembly of all the reads of the hot spring metagenome. The vertical axis shows the percentage of contigs annotated in each phylum. Different colors are used to represent the reported phylum

De-novo assembly was done with Newbler [14], Ray [16] and MetaVelvet [18] (see Table 6). Newbler assembly was set to minimum identity (mi = 95) and minimum length (ml = 60). Ray and MetaVelvet assembly software tools were configured to use 31 k-mers. Annotation, for the Large contigs (> 500 bases) reported by Newbler, was done using AMPHORA2 [17], MEGAN [23] and RAIphy [47]. AMPHORA2 and RAIphy were executed with default parameters. For MEGAN, we generated a BLASTx-comparison file of the Large contigs (> 500 bases) against a local NR in XML format (downloaded on April 2016) (see Additional file 1 and Additional file 3 for the details). Figure 7 summarizes these results.

**Table 6:** Assembler statistic reported by each tool on the original hot spring dataset, without binning

	Total large contigs (> 500 bp)	Reads assembled	Largest contig (bp)	Expected genome size (Mbp)	N50	AVG contig length (bp)	Peak depth	GC (%)
Newbler	11,739	804,983 (87%)	232,982	27	3267	2349	2.1	61
Ray	12,369	768,803 (83%)	72,115	14	1143	1134	4.8	61
MetaVelvet	17,720	797,792 (86%)	7084	19	1199	1104	2.6	61

**Fig. 8:**
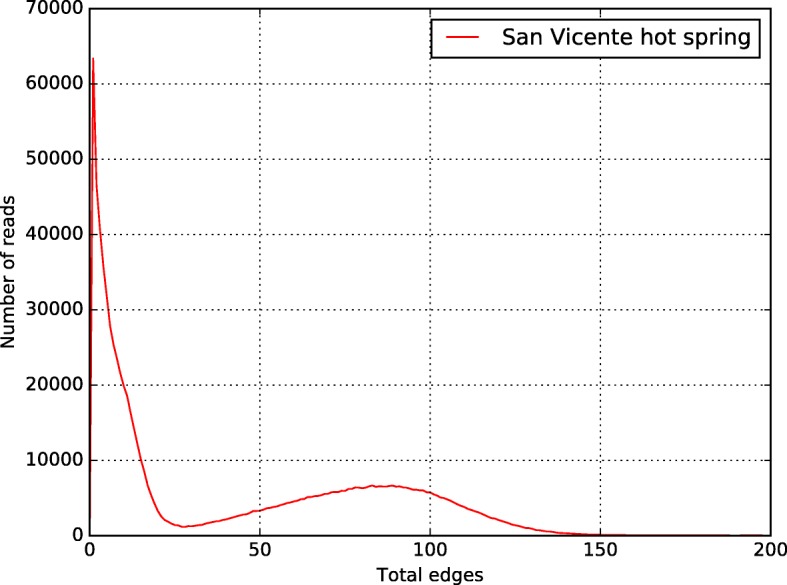
Number-of-edges histogram for the hot spring metagenome. A normal distribution can be observed on the range 30 to 150 edges

Binning process with CLAME was executed using70 bases alignment and without edge threshold restrictions. Using the Edge analysis stage, CLAME was executed again using 70 bases and restriction for the range 30 edges lower bound and 130 edges upper bound (see Fig. 8). Only the biggest bin was conserved for further analysis.

**Fig. 9:**
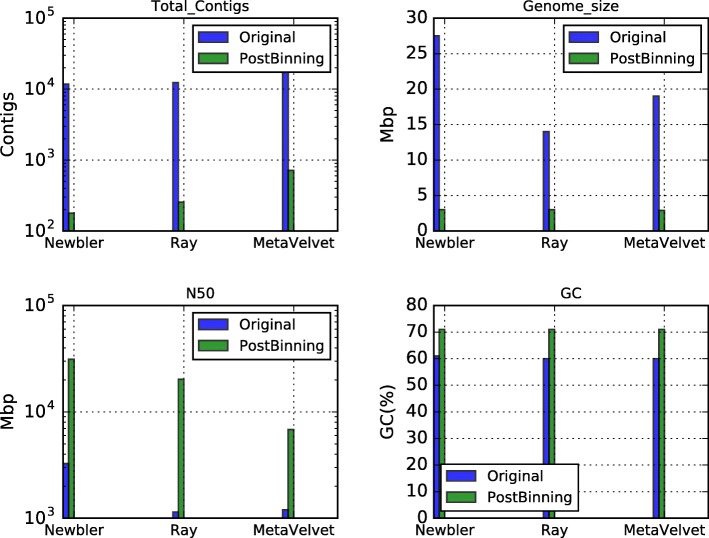
Comparative assembly of the thermal metagenome (before and after binning). The upper left graph shows the number of contigs produced using all the reads (blue bar) versus the contigs produced from CLAME’s biggest bin (green bar). The upper right graph shows the expected genome size. The lower left graph shows the N50 estimation. And the lower right graph shows the GC-percentage for the produced contigs

**Fig. 10:**
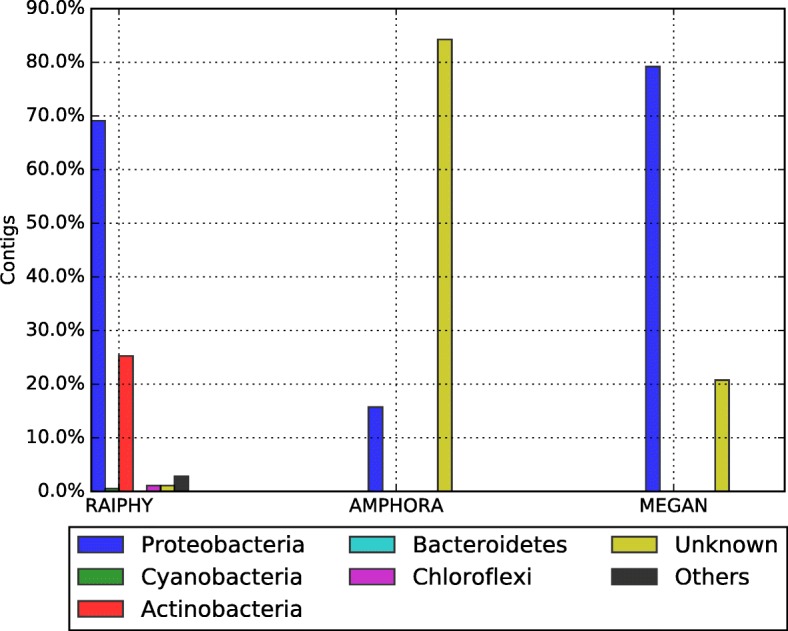
Taxonomy classification at phylum level for the 178 contigs generated by Newbler, using the reads from CLAME’s biggest bin of the thermal metagenome. The vertical axis shows the percentage of contigs annotated in each phylum. Different colors are used to represent each phylum

**Fig. 11:**
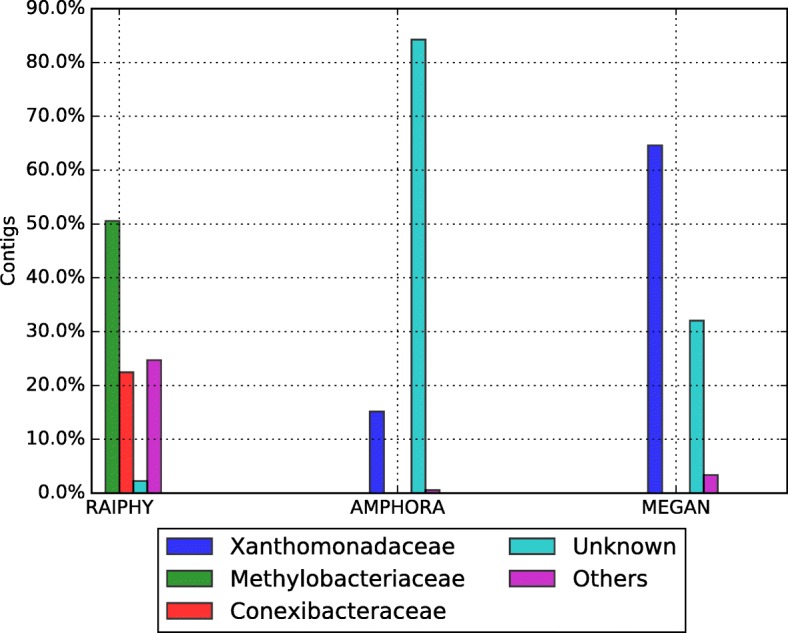
Taxonomy classification at family level for the 178 contigs generated by Newbler, using the reads from CLAME’s biggest bin of the thermal metagenome. The vertical axis shows the percentage of contigs annotated in each family. Different colors are used to represent each family

Assembly for the biggest bin was done using Newbler [14], Ray [16] and MetaVelvet [18] (see Table 7 and Fig. 9). Newbler parameters were: minimum identity 95 and minimum length 60. Ray and MetaVelvet assembly software tools were configured to use 31 k-mers. Large contigs generated by Newbler were classified with AMPHORA2 [17], MEGAN [23] and RAIphy [47] (Figs. 10 and 11). For MEGAN, we previously generated a BLASTx-XML comparison file of the Large contigs (> 500 bases). The assembly completeness for Newbler’s contigs was measured in terms of gene content and Universal Single-Copy Orthologs presence (see Additional file 1 and Additional file 2 for the details).

**Table 7:** Assembler statistic reported by each tool on the hot spring dataset of the biggest bin produced by CLAME

	Total large contigs (> 500 bp)	Reads assembled	Largest contig (bp)	Expected genome size (Mbp)	N50	AVG contig length (bp)	Peak depth	GC (%)
Newbler	178	380,796 (99%)	99,748	3.0	31,130	17,067	60	71
Ray	255	372,145 (97%)	72,110	3.0	19,598	20,242	23	71
MetaVelvet	712	371,284 (97%)	26,703	2.9	6816	4135	40	71

**Fig. 12:**
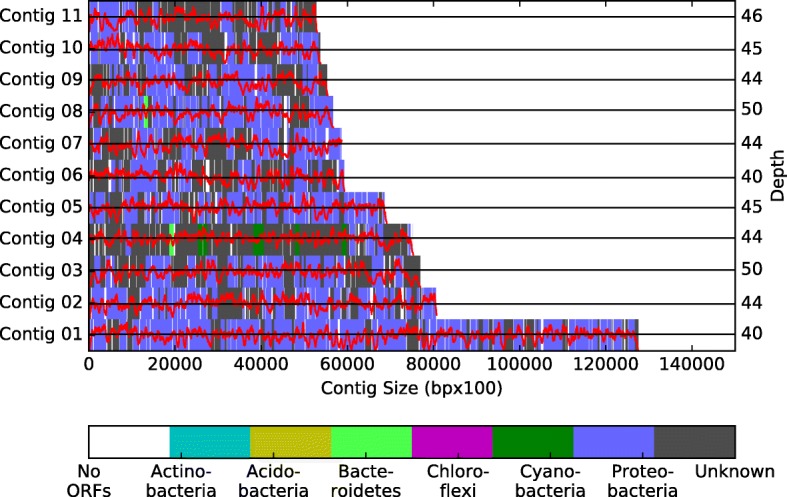
Draft-genome contig depth, open reading frames (ORFs) position and BLASTp annotation (for the eleven largest contigs). The red line illustrates the reads that align with each position of the contig. A color bar is used to illustrate the base position where each ORF is found. The bar’s color represents the achieved annotation at the phylum level. Different colors are used to represent each phylum

Putative open reading frames (ORFs) were detected using CheckM [49], Prodigal [50] and Genmark [51] tools (Table 8). Quality control for the ORFs reported by Prodigal was done using BLASTp [38] against the NR database from NCBI. Then we employed MEGAN [23] to assign each ORFs into a taxonomic level (Fig. 12). Universal Single-Copy Orthologs analysis was done using BUSCO tool [52], (see Additional file 1 and Additional file 2 for the details).

**Table 8:** Gene composition analysis for the Newbler’s Large contigs assembled of CLAME’s biggest bin of the hot spring metagenome

	CheckM	Prodigal	Genmark
Total ORFs	2726	2726	2661
Number of contigs	173	173	168
ORFs distribution	0.96	0.96	0.86

**Fig. 13:**
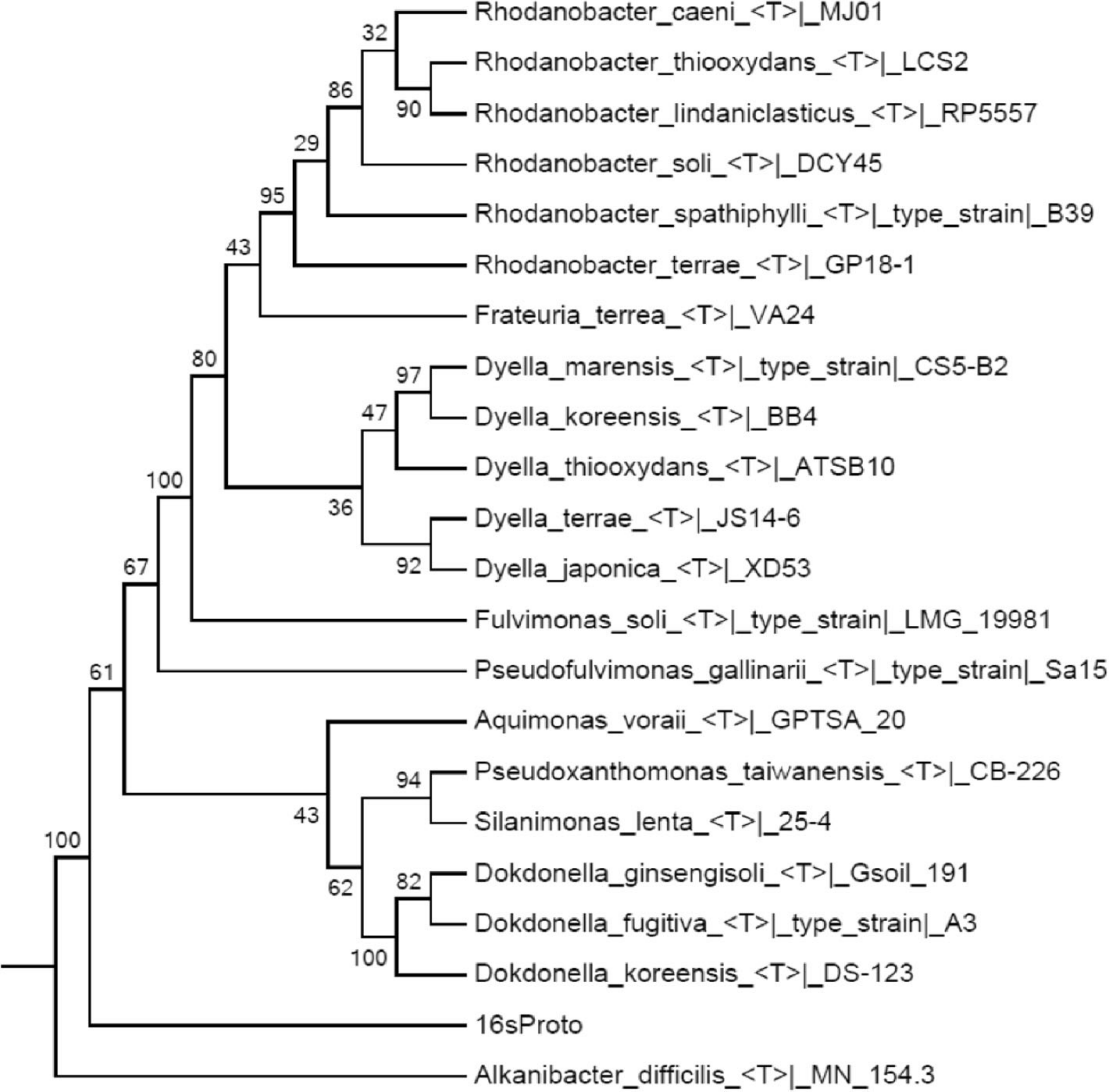
Draft-genome Phylogenetic tree inferred by using the Maximum Likelihood method with the Jukes-Cantor mode, based on our complete 16S ribosomal assembled gene (16sProto). The values in the branches indicates the percentage of replicate trees in which the associated taxa clustered together in the bootstrap test. Branches with values with less than 50% bootstrap are collapsed

Initial taxonomical classification of the organisms represented within the resultant assembled contig set was done searching contigs that contain 16S ribosomal gene sequences. The selected contigs were manually curated, annotated (Table 9) and used to build an evolutionary tree (Fig. 13). The phylogenetic tree was inferred by using the Maximum Likelihood method with the Jukes-Cantor model [53] and the process described by Brumm et al. [54]. We conserved the same number of replicates (500) and bootstrapped tree topology to represent the evolutionary history of the taxa analyzed. We used Brumm et al., strategy to obtain the initial tree(s). However, our analysis involved 29 nucleotide sequences, instead of 26 samples. There were a total of 547 positions in the final dataset. All the analysis were developed on MEGA 7.0 [55].

**Table 9:** BLASTn top 7 hits report for the 16S rRNA gene sequence found in the Newbler’s contig00154 of the assembly of CLAME largest bin of the hot spring metagenome

	Score (Bits)	Ident (%)	E-Value	Accession
Uncultured bacterium clone 16S-27F&1492R-C12-clone6	2241	99	0.0	KX348539.1
Uncultured bacterium clone B63	2228	99	0.0	AF407725.1
Uncultured bacterium clone EG90	2044	95	0.0	KC189660.1
Uncultured bacterium clone JN11	2039	95	0.0	JN868991.1
Uncultured bacterium clone LONG_SPR_11F	2026	95	0.0	KF836265.1
*Metallibacterium scheffleri* strain DKE6	1891	93	0.0	NR_118103.1
*Dokdonella koreensis* DS-123	1874	92	0.0	CP015249.1

**Fig. 14:**
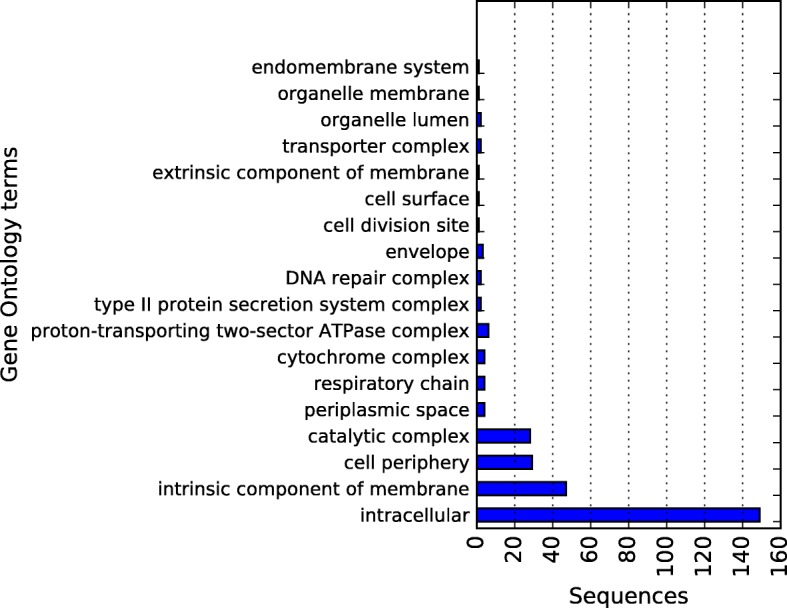
Draft-genome gene ontology annotation in the cellular component category at level 4 for the 2726 ORFs predicted by Prodigal. The horizontal axis shows the total of sequences assigned to each category

**Fig. 15:**
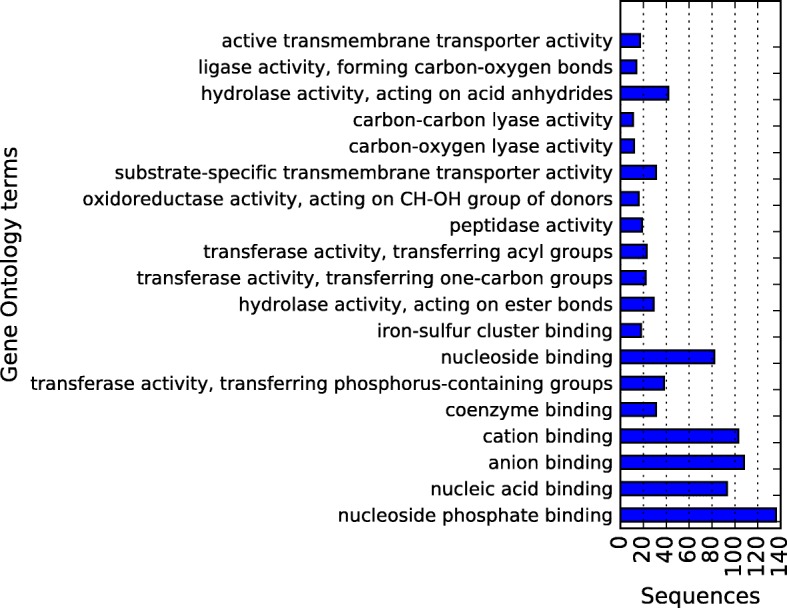
Draft-genome gene ontology annotation in the molecular component category at level 4 for the 2726 ORFs predicted by Prodigal. The horizontal axis shows the total of sequences assigned to each category

**Fig. 16:**
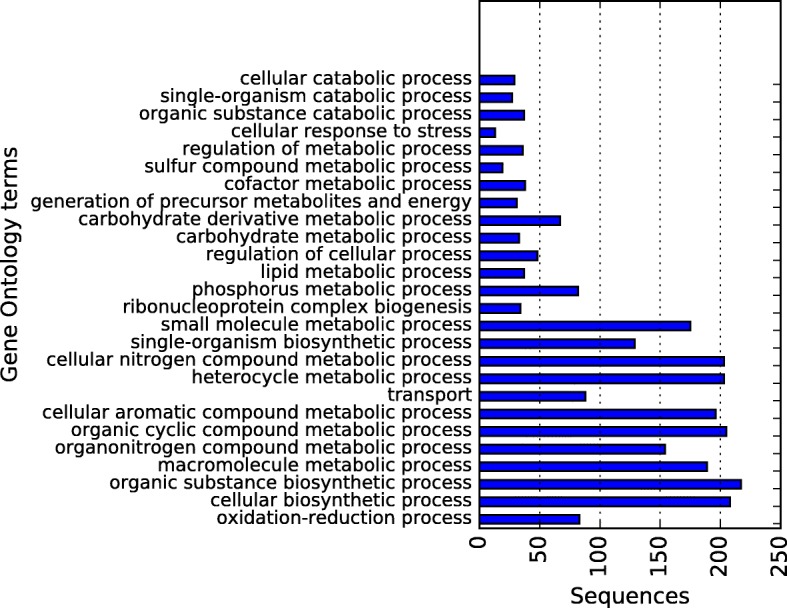
Draft-genome gene ontology annotation in the biological process category at level 4 for the 2726 ORFs predicted by Prodigal. The horizontal axis shows the total of sequences assigned to each category

In order to get an insight into the functional annotation of the predicted proteome of the *Xanthomodaceae* of the San Vicente Hot spring, Gene Ontology annotation was performed for the 2726 ORFs predicted by Prodigal (Figs. 14, 15 and 16). It was done using BLASTp comparisons of all the predicted peptides against the NCBI’s protein NR database and BLAST2GO version 2.8 [56] annotation tool. Additionally KAAS (KEGG Automatic Annotation Server) [57] was employed to provide a detail functional annotation of predicted genes.

We compared CLAME against MetaBinG [27], MetaProb [28], BiMeta [29], and AbundanceBin [31] tools. For the tools in which the number of bins or species can be specified, we decided to set it to 5, according the number of phyla found by the annotation tools described previously. The biggest bins reported by each tool were assembled using Newbler [14], it was setting at minimum identity (mi = 95) and minimum length (ml = 60) in all the cases. Table 10 compares these results versus CLAME’s de-novo assembly for the biggest bin.

**Table 10:** Newbler assembly statistics of the bins reported by each tools on the hot spring metagenome. It also shows the time it took each tool to create the bins

Tool	Total Bins	Total reads	Large contigs	Expected genome size (Mbp)	AVG contig length (bp)	Largest contig (bp)	N50	GC (%)	Time (m)
CLAME	2	380,846	178	3.03	17,067	99,748	31,130	71	9
		446	24	25,157	1048	2791	1054	66.17	
BiMeta	5	113,070	2131	2.3	1082	28,701	1077	65	211
		22,877	728	0.6	867	6907	860	38	
		273,565	995	2.98	3002	49,922	11,620	72	
		283,509	3499	5.95	1701	45,994	2185	70	
		207,349	3857	9.73	2523	41,372	4961	51	
MetaProb	5	275,160	3423	5	1460	53,631	1561	69	21
		60,580	1350	1.3	966	11,767	966	58	
		204,718	4262	9.45	2217	29,837	4059	51	
		47,618	766	0.7	901	6858	898	61	
		312,294	1486	4.7	3149	63,982	6146	72	
AbundanceBin	3	459,353	950	3.7	3876	75,296	12,564	69	1063
		190,112	6574	8.1	1240	8964	1475	56	
		250,905	8938	8.6	968	4762	1005	62	
MetaBinG	3	521,865	7765	9.8	1253	30,729	1278	66	131
		212,100	3115	4.6	1480	9988	1829	71	
		125,979	4764	6.7	1400	13,502	1647	51	

We also analyzed the other bins (with at least 2000 reads) produced by CLAME. These bins were assembled with Newbler [14], minimum identity (mi = 95) and minimum length (ml = 60), and annotated with AMPHORA2 [17], MEGAN [23] and RAIphy [47]. AMPHORA2 and RAIphy were executed with default parameters and for MEGAN we generated a BLASTn-comparison file of the Large contigs (> 500 bases) against a local NT (downloaded on May 2017) in XML format (see Additional file 1 for the details).

**Fig. 17:**
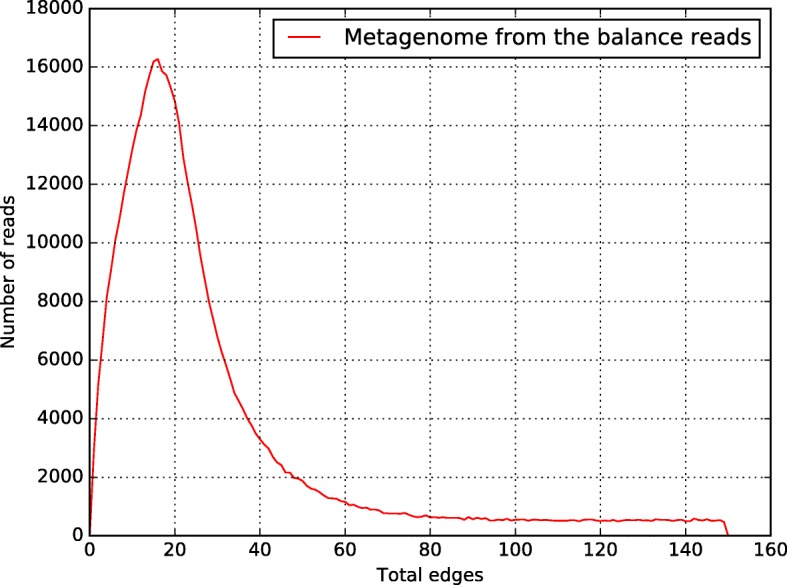
Number-of-edges histogram for the thermal metagenome from the balance reads (without the reads used for the draft genome). A normal distribution can be observed on the range 10 to 20 edges

In order to study the other species presents in the metagenome, we elaborated an auxiliary dataset by deleting the reads binned in the first CLAME execution and conserved the balance of the read in the original dataset. A total of the 519,524 reads conform this second dataset. CLAME was executed on this dataset using 15 bases matching and edge thresholds for the range 10 to 20 (Fig. 17), only bins with at least 2000 reads were reported. The parameters were configured experimentally to get suitable bins. The biggest bin produced by CLAME was assembled with Newbler [14] and annotated using AMPHORA2 [17], Megan [23] and RAIphy [47] (Tables 11 and 12). AMPHORA2 and RAIphy were executed with default parameters. For MEGAN we generated a BLASTn-comparison file of the Large contigs (> 500 bases) against a local NT (downloaded on May 2017) in XML format.

**Table 11:** Thermal metagenome Newbler assembler statistics for the balance reads (without the reads used for the draft genome)

Bin number	Total of reads	Total large contigs	Largest contig (bp)	Expected genome size (Mbp)	N50	AVG contig length (bp)	GC (%)
0	146,977	5056	8852	5.9Mpb	1277	1163	51.58

**Table 12:** Annotation of Newbler’s Large contigs assembled from the thermal metagenome from the balance reads (without the reads used for the draft genome)

Phylum	MEGAN	RAIphy	AMPHORA 2
Cyanobacteria	3214 (63.57%)	3339 (66.04%)	37 (0.73%)
Proteobacteria	167 (3.30%)	1161 (22.96%)	2 (0.04%)
Bacteroidetes	18 (0.36%)	36 (0.71%)	2 (0.04%)
Others	411 (8.13%)	520 (10.28%)	1 (0. 019%)
Unknown	1246 (24.64%)	0 (0.00%)	5014 (99.17%)

### CLAME computational performance

**Fig. 18:**
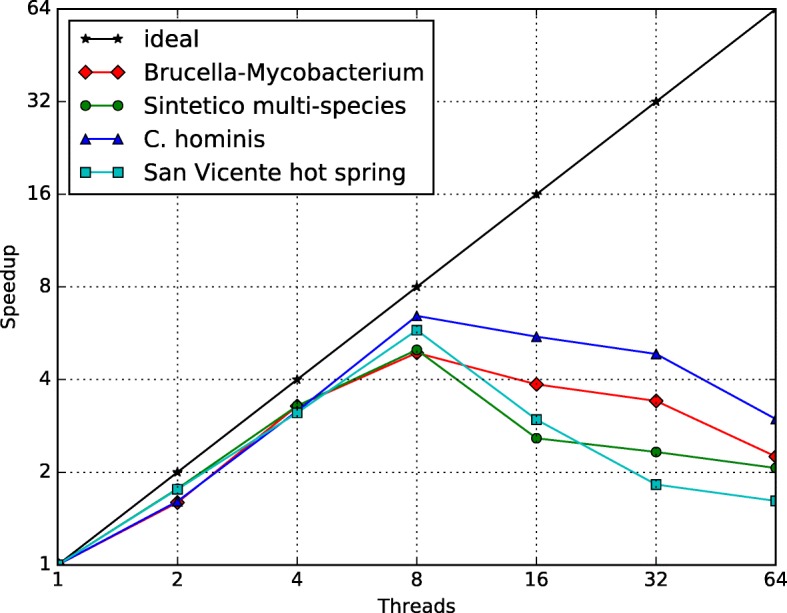
CLAME speed performance. The horizontal axis shows the number of threads used. Vertical axis shows the speedup with respect to the 1 thread execution

**Fig. 19:**
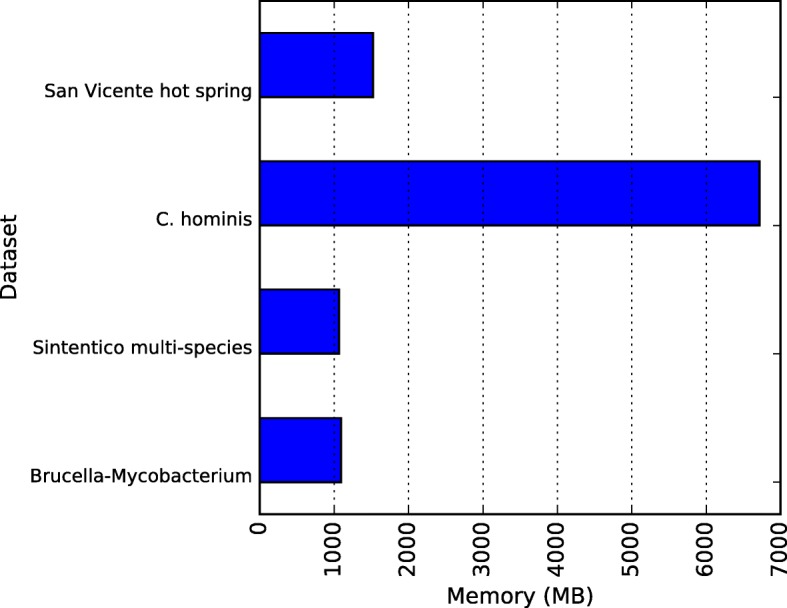
CLAME memory performance. The horizontal axis shows the RAM memory usage for each dataset

We show CLAME’s speed and memory performances on Figs. 18 and 19. All the experiments were performed on a computer equipped with 64 Intel(R) Xeon(R) CPU X7560 @ 2.27GHz and 500 GB of RAM. CLAME was implemented in C ++ using OpenMP (Open Multi-Processing) interface. We executed CLAME employing 1, 2, 4, 8, 16, 32 and 64 threads on each dataset previously explained. We selected the best of five executions. Valgrind [58] was used to measure CLAME’s memory usage. We took the maximal memory usage of each experiment.

## Results

We calibrated CLAME using public available NGS data of 454 and Illumina MiSeq platforms, then we used it to study the metagenomic dataset obtained from a hot spring in the Colombian Andean Mountains (located in San Vicente, Risaralda, Colombia).

### Simulated metagenome

We tested CLAME with the simulated metagenome, which was created combining DNA sequencing from *Brucella canis* and *Mycobacterium tuberculosis*. The mixed data set, of 665,039 reads, was elaborated, as described in the methods section, using 289,917 reads of *B. canis* and 375,122 reads of *M. tuberculosis*. In order to understand the profile of the number of edges, we ran CLAME three times: only with *M. tuberculosis* reads, only with *B. canis* reads, and with the simulated metagenome (the combination of both). Figure 2 illustrates the number of edges histogram, produced by CLAME in the read alignment stage using 70 bases alignment. CLAME generated two main bins that contained 353,876 and 280,014 reads. The first bin, with 353,876 reads, was formed exclusively by reads of *M. tuberculosis*; they represent 94.3% of the original *M. tuberculosis* set. The second bin, with 280,014 reads, was composed exclusively by *B. canis* reads. They represent 96.5% of the original *B. canis* read set. Most of the remaining reads were short (smaller than 70 bases) and therefore they were binned as singletons.

We compared CLAME’s performance against the other binning tools. Table 1 summarizes the results produced by CLAME, MetaBinG [27], MetaProb [28], BiMeta [29], and AbundanceBin [31]. It shows that although most tools produced individual bins for *B. canis* and *M. tuberculosis* reads, only CLAME created bins that contained reads from only one species. The table also shows the time it took each tool to create the bins, (all the tools were executed on one thread), and it shows that CLAME is the fastest of all.

### Simulated multi-species metagenome

Using the biological information from the San Vicente hot spring, explained in detail in the next section, we elaborated a synthetic metagenome that can simulate the diversity found in that metagenome. We selected five species from the NCBI database and elaborated a synthetic metagenome as is described in the methods section (see Table 2).

CLAME was tested using this synthetic multi-species metagenome. In order to understand the number of edges profile, we ran CLAME using 70 bases alignment and without edge thresholds. Figure 3 shows the histogram produced, it can be seen that the *Gammaproteobacteria* has the major contribution while the Cyanobacteria and the other species are present in lower proportion. It also shows that the *Gammaproteobacteria* has less than 50 edges. The *Actinobacteria* is the one with an average higher number of edges. The bins produced by executing CLAME with edge thresholds for the range (1, 50) is shown in Table 3. The table shows the number of bins produced by CLAME and the contribution of each species into the reported bins. We observe that CLAME binned 65% of *Gammaproteobacteria* reads into 4 bins. The biggest bin contains 209,642 reads (the 56% of the reads belong to the *Gammaproteobacteria*). From there it is possible to observe that CLAME recovers most part of the predominant species. CLAME also recover the 35% of the Cyanobacteria in 2 bins and 65% of the *Actinobacteria* in a single bin. It is important to note the sensibility of CLAME to bin the reads without mixing reads from different species.

We compare CLAME’s results against MetaBinG [27], MetaProb [28], BiMeta [29], and AbundanceBin [31]. Table 3 shows the results of each tool, the number of bins and their size. CLAME was the fastest of all, and the only that doesn’t combine reads from more than 1 species in each bin. These results show CLAME’s ability to separate reads from closely related species, even if the species are of the same class.

### Illumina MiSeq metagenomic read set

To test CLAME with a real dataset, we used a partially annotated metagenome recovered from human feces. The metagenome comprises 9,052,596 Illumina pair-ended reads that were generated in one study focused on the intestinal protozoan parasite *Cryptosporidium hominis* [45]. The study reports that a total of 1,066,460 metagenome pair-ended reads belong to *C. hominis*. We took the raw reads and prepared the dataset according to the process described in the methods section. After filtering and merging the reads, 6,052,596 reads were included in the analysis, from those, 728,439 reads were *C. hominis*.

CLAME was executed using 100 bases alignment with the complete metagenome. Figure 4 shows the histogram of the number of alignments of the metagenome (in red) and the distribution of only *C. hominis* reads (in blue). Note that *C. hominis* reads follow a normal-like distribution in the range 15 to 100 edges. Consequently, CLAME was configured with two thresholds, at 100 and 15 edges. It reports 731 bins with at least 500 reads. We found that 407 of those bins were formed exclusively by reads from *C. hominis*, for a total of 467,939 merged reads. Those reads were (64%) of the reads reported in the respective paper [45] as *C. hominis* reads. Bowtie2 [46] reported a 99.72% overall alignment rate to the respective *C. hominis* reference genome. Figure 5 illustrates the coverage of the reads of those bins (blue line) on the whole *C. hominis* genome, and the reads reported as *C. hominis* on the paper (red line). The remaining *C. hominis* reads (the 36%) were found in 1611 bins with less than 500 reads.

Moreover, we analyzed the two main bins produced by CLAME. Table 4 shows Newbler de-novo assembly for the main bins. The biggest bin produced by CLAME contains 932,332 reads. It reports 3211 Large contigs; annotation of these contigs using AMPHORA2 [17], MEGAN [23] and RAIphy [47] indicated that these contigs belong to *Veillonella* bacteria (Table 5). The second biggest bin produced contains 514,053 reads. It produced 447 Large contigs; annotation of these contigs using AMPHORA2 [17], MEGAN [23] and RAIphy [47] indicated that these contigs belong to *Bifidobacterium* bacteria (Table 5).

### San Vicente hot spring

The waters of the San Vicente hot spring showed a relatively low count of total cells (2 × 104 cells/mL) and we could only recover a total of nine Cyanobacteria and eight heterotrophic-thermophilic morphotypes using traditional culture approaches. After the enrichment of the sample at 45 °C for 15 days, we observed that a filamentous *Cyanobacterium* dominated the community; although several small cells were also present (Fig. 6) suggesting that the desired reduction in the complexity of the community was achieved.

DNA extraction and metagenome sequencing based on this enriched sample, which is described in the methods section, generated 926,130 reads that were cleaned and assembled with commonly used software and we obtained 900,370 quality reads. Table 6 presents the general statistics of the contig set assembled by Newbler [14], Ray [16] and MetaVelvet [18] for these reads. It illustrates that Ray produced the best peak depth, however, it reported a greater number of contigs than Newbler. MetaVelvet generated the highest number of contigs and the lowest average contig length. Newbler had the best N50 statistic on a low number of contigs (with more than 500 bases), all of them with a peak depth greater than two.

We took Newbler’s contigs with more than 500 bases for further analyses; we explored them with the metagenomic annotation pipelines AMPHORA2 [17], MEGAN [23] and RAIphy [47]. Figure 7 summarizes the results. For all the tools, the Proteobacteria was the predominant phylum while Cyanobacteria, Actinobacteria, Bacteroidetes, Chloroflexi are present in lower abundance. RAIphy assigns all contigs on a single phylum, however, manual inspection showed a large number cases that contradict the other tools. For AMPHORA2 the unknown classification was the most frequent, leaving most of the contigs without any taxonomical assignation. This is expected since AMPHORA2 is focused on detecting 33 bacterial genes that are very useful for taxonomical purposes. MEGAN is the one that classifies more contigs (20%), however, the number of contigs annotated in each phylum was not enough to propose a draft genome.

The original dataset, after cleaning, was binned using CLAME. Figure 8 shows the edge histogram, produced by CLAME, considering 70 bases alignment. It shows a normal-like distribution in the range of 30 to 130 edges. Consequently, we ran CLAME with these parameters. CLAME reported a total of 11 bins with at least 2000 reads.

The biggest bin is composed by 380,846 reads (42.3% of the total of reads). Table 7 summarizes the number of contigs and characteristics of the assembly of these reads using Newbler [14], Ray [16] and MetaVelvet [18]. Figure 9 compares these results versus the original assembly without the binning step. We can conclude that the number of contigs decreased compared with the initial assembly.

The 178 large contigs (> 500 bp), produced by Newbler, were classified with AMPHORA2 [17], MEGAN [23] and RAIphy [47]. Figure 10 shows that Proteobacteria is the predominant phylum for all the tools, while the other phyla have almost disappeared if we compare it with the classification of the whole metagenome on Fig. 7. Figure 11 shows that most contigs were assigned to the *Xanthomonadaceae* family. We used these contigs to produce the Colombian thermophile *Xanthomonadaceae_UdeA_SF1* draft genome (available in CLAME’s GitHub and on the NCBI’s project PRJNA431299).

Table 8 summarizes, CheckM [49], Prodigal [50] and Genmark [51] tools report. On the contigs assembled from the biggest bin, there are more than 2600 open reading frames (ORFs) that codify as possible genes, and since there are 3.0 Mbp on the genome, it indicates that there is close to 1 coding region per Kbp.

An additional quality control was done using MEGAN [23] to assign each ORFs into a taxonomic level. As it is shown in Fig. 12, for the first 11 largest contigs, most of the ORFs were annotated as Proteobacteria (blue bars in the graph). Moreover, we measured the assembly completeness in terms of gene content by means of Universal Single-Copy Orthologs using BUSCO [52] tool. We found 32 of the 40 (80%) essential genes reported by BUSCO were found in the proposed draft genome. Using the set of standards for the minimum information regarding a metagenome-assembled genome (MIMAG) proposed by Bowers et al. [59] and the previous results, we can conclude that we introduce a High-quality draft genome.

Analysis of the complete 16S ribosomal gene, which was contained in the contig00154, using BLAST [38] against the NT database from the NCBI, indicates that our 16S sequence is related to an uncultured bacterium clone B63 recovered from Australia’s Great Artesian Basin. The top 7 of the BLASTn results are shown in Table 9. To refine the query, we reconstructed the 16S phylogeny using the Ribosomal Data Project database [60] as a curated reference, the Maximum Likelihood method based on the Jukes-Cantor model [53] and the Brumm et al. [54] process. It showed that our sequence is closely related to several uncultured bacteria within the family *Xanthomonadaceae* of the *Gammaproteobacteria*. Besides, the phylogeny reconstructed only based on culture-type strains showed that the obtained 16S sequence is consistently within Order *Xanthomonadales*, separated from the outgroup *Alkanibacter difficilis* Order *Sinobacteriales* and apart from the cluster composed by the Genus *Dokdonella* and other *Xanthomonadales* such as *Rhodanobacter*, *Dyella*, *Aquimonas* and *Pseudoxanthomonas* (Fig. 13).

Gene Ontology annotation for the 2726 ORFs predicted by Prodigal tool, using BLASTp [38] comparisons and BLAS2TGO [56], indicates that 94 % of the predicted peptides exhibited a hit with an E-value below the threshold of 1E-5; and that only 668 proteins could be finally annotated with at least a GO term.

Figures 14, 15 and 16 present the obtained results of Gene Ontology annotation at level 4 for cellular component, molecular function, and biological process. According to cell localization prediction, most of the proteins were assigned to the intracellular space, while others were localized to different components such as cell membrane, periplasmic space, and macromolecular complexes. The Molecular function prediction shows, at the top, the category organic substance biosynthetic process, followed by anion and cation binding. Nevertheless, other categories like hydrolases, transferases, transporters, peptidases, ligases, and lyases were well represented. For the biological process annotation, 26 terms were assigned for 2581 hits, being organic substance biosynthetic process at the top of the list with 217 hits. Also above 200 hits were, in order of abundance, cellular biosynthetic process, organic cyclic compound metabolic process, heterocycle metabolic process and cellular nitrogen compound metabolic process.

Moreover, using the KEGG pathway annotation tool KAAS [57] we were able to confirm that the glycolysis, pentose phosphate, Glyoxylate, Fatty acid biosynthesis, beta-Oxidation and TCA cycle enzymes were all present and complete. For the genetic information processing complexes, RNApol beta subunits, as well as alpha and omega, were annotated, but the delta was missed in our putative protein set. In the case of DNA replication, bacterial subunits of the holoenzyme pol III were detected except for the psi and theta subunits. Helicase, primase, SSB, DNA ligase and RNAses HI, and HII were also annotated by KAAS. Within the homologous recombination system, RecA and RecJ proteins were also missed by the annotator. Two-component systems were also annotated; the family OmpR was the most frequent with the histidine kinases PhoR, PhoQ, CreC, CusS, and ArcB. The second most frequent was the NarL family with orthologous for the kinases BarA, DesK, and VraS.

CLAME binning for the hot spring metagenome was compared with the results of MetaBingG [27], MetaProb [28], BiMeta [29], and AbundanceBin [31]. Table 10 shows the number of Large contigs (size > 500 bases) and the genome size estimation produced by Newbler de-novo assembly of all binning tools. We included the same fields for the de-novo assembly of CLAME’s biggest bin. It produced fewer contigs than the assembly of the others tools-results. Moreover, the genome size estimation is the closest to the expected bacteria genome-size.

The additional bins (with at least 2000 reads) produced by CLAME were assembled using Newbler [14] and annotated with AMPHORA2 [17], MEGAN [23] and RAIphy [47]. AMPHORA2 doesn’t report hits because no marker can be found in these reads. MEGAN and RAIphy results indicate that the reads can be an additional part of the Proteobacteria.

Finally, removing the reads used for the draft genome, a total of 519,524 reads were left. Experimentally, we noted that using 15 bases alignment, produce a big bin with mainly a single species. The edges-histogram is illustrated in Fig. 17, and it shows that in the range 10 to 20 edges there is a second normal-like histogram. Configuring CLAME with this thresholds, it produces a bin with 146,967 reads. Table 11 illustrates that the de-novo assembly for these reads, using Newbler [14], produces 5056 contigs. We annotated these contigs using AMPHORA2 [17], MEGAN [23] and RAIphy [47]. Table 12 summarized these results and indicate that they are classified mainly as Cyanobacteria. This results coincide with the spring-water biological description made previously. Contamination can be explained by the reduce number of bases used for the overlap detection stage.

### CLAME computational performance

Figure 18 shows a close to lineal scalability of CLAME up to 8 threads. It shows that the best performance for 8 cores is obtained by the *C. hominis* dataset because it has the largest number of reads. Initial experiments have showed that the speedup is limited by the suffix tree generation, which is a sequential process. Figure 19 shows the memory usage of each experiment. It can be seen, as expected, that the usage increases with the dataset size.

## Discussion

There are few research publications that propose a new species draft genome extracted from a metagenome. Probably the main reason is that it is not a simple task. However, in many projects, the researchers are not interested in getting a genome but just testing the presence of different species. It has been known that a binning step is desired on metagenome studies. In this work, we show that CLAME can bin reads fast and efficiently. By being very strict, allowing only long and perfect alignments, and given the user thresholds, CLAME creates bins of reads from a single DNA chromosome. Furthermore, most reads are assigned to a bin despite using a very restricted alignment. We showed that the other binning tools were not very effective classifying the metagenome reads that we were analyzing. CLAME allowed us to extract most reads of a novel *Xanthomonadaceae* bacterium from a hot spring metagenome on a single bin. We validate the draft genome using several tools.

Given its speed and performance, we present CLAME as a metagenome-binning tool. CLAME works best for bacterium genomes that are well covered on the metagenome and it mainly extracts the most abundant species. For closely related species in a metagenome, but with a significant difference in concentration, the user can adjust thresholds to bin them in different groups.

In metagenomes with highly represented complex species, like in the eukaryote *C. hominis* example, CLAME created bins with mainly single species reads, but it generated too many bins. Due to the complexity of the *C. hominis* genome, the authors of the original paper used reads from two platforms (with different characteristics) to assemble it. We show that CLAME can recover reads that cover most of the protozoan parasite genome. CLAME can be used to quickly create bins of reads that can be further assembled, reducing the processing time, the risk of chimeric contigs and obtain better N50 stats.

Given the complexity of metagenomes, the different sequencing methodologies, and the variable error rates of the sequencing, it is difficult that a tool automatically creates the bins. For this reason, in CLAME we added several configuration parameters that allow the user to tune it for the particular experiment. We used CLAME to bin the reads of the most abundant species of metagenome from a hot spring.

The assembly of the biggest CLAME bin generated the draft genome presented. According to the 16S rRNA gene phylogenetic analysis, it corresponds to a novel taxon within the Order *Xanthomonadales*. Although the closest related sequences within the Ribosomal Database Project are uncultured bacteria, the phylogenetic reconstruction, including only isolated-type strains, clearly shows that our genome is within the family *Xanthomonadaceae*, close to *Dokdonella spp*. but separated from the family *Sinobacteriacea* (i.e. outgroup *Alkanibacter difficilis*). We propose it as a partial draft genome of novel thermophile *Xanthomonadal*.

The de-novo assembled genome is around 3 Mb, with 2726 predicted ORFs; it is a small genome size compared to *Dokdonella* and *Dyella*, both with genomes around 4.5 Mb and 3519 and 3966 annotated proteins, respectively. Our BUSCO annotation results show that the genome is not complete, 32 genes of 40 essential were found. The KEGG annotation pipeline further confirmed this, since the subunits RNApol and DNApol were not completely present in our annotated contigs. However, the main metabolic pathways such as TCA, glycolysis, pentose phosphate, Glyoxylate, Fatty acid biosynthesis and beta-Oxidation were present and they were completely annotated by the same database. In addition, proteins from all different cell localizations were annotated. BUSCO estimation of 80% completeness of the genome might be adequate as a reference lower limit, although we cannot predict if the genome size of the novel Andean *Xanthomonadaceae* is as big as the *Dyella* and *Dokdonella* genomes.

The global genome annotation did not show any special adaptations of this prokaryote; its metabolic profile is very similar to the other organism of this Family, where we can find a Heterotrophic lifestyle living at expenses of the Cyanobacteria that shared the thermal water.

## Conclusions

While several metagenomic binning tools were unable to separate the synthetic and real problems that we proposed, we show that CLAME was faster and better on these problems. CLAME is a tool that helps researchers to analyze metagenomes by creating bins of reads that belong to a single DNA chromosome, without the need of a reference genome. This is important since most of the unculturable microorganisms do not have reference genomes. Therefore, it can be used to improve metagenome analysis by grouping reads from DNA fragments of novel species, such as the *Xanthomonadal* presented in this work. This draft genome is one of the first thermophile members of this family, and it was possible to obtain thanks to CLAME.

## Additional files

Additional file 1: Detail description for the all experiments execution. (DOCX 25 kb)

Additional file 2: Full table list . (DOCX 26 kb)

Additional file 3: Full figures file. (PDF 2788 kb)

## Abbreviations

Bp: Base pair
CLAME: From the Spanish “CLAsificador MEtagenomico”
CNSG: Centro Nacional de Secuenciación Genómica
CPU: Central processing unit
DNA: Deoxyribonucleic acid
FM: Full-text minute-space
GB: Gigabytes
KAAS: KEGG Automatic Annotation Server
KEGG: Kyoto Encyclopedia of Genes and Genomes
MASL: Meters above sea level
MCL: Maximum Composite Likelihood
NCBI: National Center for Biotechnology Information
NGS: Next Generation Sequencing
NJ: Neighbor-joining
NR database: Non-redundant database
ORF: Open Reading Frame
pH: Potential of hydrogen
RNApol: Ribonucleic acid polymerase
TCA: Tricarboxylic acid cycle
