# Supplementary material for: CLAME: a new alignment-based binning algorithm allows the genomic description of a novel Xanthomonadaceae from the Colombian Andes
Source: BMC Genomics. 2018 Dec 11;19(Suppl 8):858. doi: 10.1186/s12864-018-5191-y (PMC6288851; doi:10.1186/s12864-018-5191-y)
Supplement: Supplementary file 1 — Detail description for the all experiments execution. (DOCX 25 kb) [file 12864_2018_5191_MOESM1_ESM.docx]

**CLAME: Supplementary-Material document**

Benavides A^1*^, Isaza JP^2,4^, Niño-García JP^3^, Alzate JF^2,4^ and Cabarcas F ^1,2^

* Correspondence: bernardo.benavides@udea.edu.co

1 Grupo SISTEMIC, Ingeniería Electrónica, Facultad de Ingeniería, Universidad de Antioquia UdeA; Calle 70 No. 52-21, Medellín, Colombia

Full list of author information is available at the end of the article

**Detail Methods.**

Simulated simple metagenome

A synthetic metagenome dataset was created using 289,917 reads of *Brucella canis* and 375,122 reads of *Mycobacterium tuberculosis,* both generated with the ROCHE’s 454 titanium platform and associated with the NCBI´s bioprojects PRJEB4803 and PRJEB8877, respectively. The reads were quality trimmed at Q30 using Prinseq [43] (prinseq-lite.pl -fastq brucella.fastq -out_format 3 -no_qual_header -out_good cleaned_brucella_reads -out_bad null -min_len 50 -rm_header -trim_qual_right 30 -trim_qual_type min) (prinseq-lite.pl -fastq mycobacterium.fastq -out_format 3 -no_qual_header -out_good cleaned_mycobacterium_reads -out_bad null -min_len 50 -rm_header -trim_qual_right 30 -trim_qual_type min). The cleaned reads were concatenated on a simple multi-fasta file to get a total of 665,039 mixed reads that formed the *Brucella*-*Mycobacterium* synthetic metagenome. These reads were binned using CLAME, with at least 70 bases alignment (clame -multiFasta Brucella_Mycobacterium.fasta -b 70 -output brucellaMycobacterium -print). The parameters were determined experimentally, such that CLAME generated 2 bins for this metagenome.

*B. canis* and *M. tuberculosis* number of edges histogram is shown in Figure 2, it was plotted with the in-house Python script plotHist.py (plotHist.py -f brucellaMycobacterium.links -c 1 -f2 brucella.links -c2 1 -f3 mycobacterium.links -c3 1 -line -label1 Metagenome -label2 'B. canis' -label3 'M. tuberculosis' -lu 150); this script can be found as part of CLAME. Quality control for each bin was checked, by matching the content (read codes) of each bin against the original fastq files.

We also used MetaProb [28], BiMeta [29], AbundanceBin [31] and MetaBinG [27] tools to bin the metagenome. (BiMeta -finput Brucella_Mycobacterium.fasta -numSp 2 -l 30 -m 15 -ssize 20 -read 0), (MetaProb -si Brucella_Mycobacterium.fasta -numSp 2 -feature 2 -m 45), (abundancebin -input Brucella_Mycobacterium.fasta -output Brucella_Mycobacterium_Bin -bin_num 2), ((CPU version) metabing Brucella_Mycobacterium.fasta > Brucella_Mycobacterium_Bin.txt). For the tools in which the number of bins or species can be specified, this parameter was set up to 2. Quality control for each tool was checked, by matching the content (read codes) of each bin against the original raw files. Table 1 shows the results of all the binning tools.

Simulated multi-species metagenome

We created a metagenomic dataset based on the bacterial genomes of five species which were downloaded from the NCBI database: *Synechocystis*, SRA code DRR106442, Cyanobacteria; *Dokdonella*, SRA code SRR4217676, Proteobacteria *Gammaproteobacteria*; *Hymnobacter*, SRA code SRR1334914, Bacteroidetes *Cytophagia*; *Microbacteria*, SRA code SRR5493999, Actinobacteria *Actinobacteria*; and *Rhizobium*, SRA code SRR5165471, Proteobacteria *Alphaproteobacteria*. For each species, the raw reads downloaded were merged into an extended single multifasta file using the Flash tool [44] (minimal identity parameter of 65 bases). In order to simulate different abundance levels, similar to the real spring-water metagenome, different amounts of extended reads were randomly taken from each dataset. Table 2 shows: the number of raw reads, the taxonomy of each species, the number of reads used (after using Flash to join read pairs), the size of the genome reported and the depth of each genome used. The final dataset was produced by concatenating the selected sequences into a single multifasta file.

CLAME was executed using 70 bases alignment and no edge thresholds (clame -multiFasta synthetic.fasta -b 70 -output clame_synthetic -print). The number of edges histogram is shown in Figure 3 (generated with the script plotHist.py -f clame_synthetic.links -c 1 -f2 cyano.links -c2 1 -f3 Dokd.links -c3 1 -f4 Hymero.links -c4 1 -f5 Mico.links -c5 1 -f6 Rhiz.links -c6 1 -line -lu 200). Using the histogram CLAME was executed again using 70 bases and edge thresholds for the range 1, 51, 10000 (clame -multiFasta synthetic.fasta -b 70 -e 1,51,1000 -output clame_synthetic -print). Quality control for each bin was manually checked, by matching the bins content versus the read codes from the original raw files.

We also executed MetaProb [28], BiMeta [29], AbundanceBin [31] and MetaBinG [27] tools with this metagenome. (BiMeta -finput synthetic.fasta -numSp 5 -l 30 -m 15 -ssize 20 -read 0), (MetaProb -si synthetic.fasta -numSp 5 -feature 2 -m 45), (abundancebin -input synthetic.fasta -output syntheticBin -bin_num 5), ((CPU version) metabing synthetic.fasta > syntheticBin.txt). For the tools in which the number of bins or species can be specified, this parameter was configured to 5. Quality control for each tool was again checked, by matching the content of each bin against the original raw file codes. Table 3 compares these results versus CLAME’s results.

Illumina MiSeq metagenomic read set

This dataset corresponds to a metagenomic sequencing experiment of human intestinal microbiota after a separation stage, where the intestinal protozoa *Cryptosporidium hominis* was enriched [45]. The original pair-ended reads cover the whole genome of this protozoan parasite, which is contained in 8 chromosomes. The reported reads belonging to *C. hominis* (1,066,460) were downloaded from SRA database Accession ERX1047563. The metagenome raw reads (9,052,596) (available in CLAME’s) were trimmed, using a minimum quality cutoff of Q30 using Prinseq [43] tool (prinseq-lite.pl -fastq crypto1.fastq -fastq2 crypto2.fastq -out_format 3 -no_qual_header -out_good cleaned_crypto_reads -out_bad null -min_len 50 -rm_header -trim_qual_right 30 -trim_qual_type min). Then the reads were merged into an extended single multifasta file using the Flash [44] tool (flash -m 65 -t 20 -o cleaned_Extended_reads.fastq cleaned_crypto_reads1.fastq cleaned_crypto_reads2.fastq). There were 6,052,596 left after these steps.

The 6,052,596 reads were binned using CLAME with 100 bases alignment and custom edge thresholds, (clame -multiFasta cleaned_Extended_reads.fastq -b 100 -e 15,101,10000 -output crypto -print -fastq). The distribution of the number of edges on the metagenome and the *C. hominis*' read contribution was plotted using the python script plotHist.py (Figure 4), (plotHist.py -f crypto.links -c 1 -line -lu 1000). We manually selected the bins that included reads from *C. hominis* genome.

CLAME performance was measured using as a control the *C. hominis* genome reference (SRA Accession ERX1047563) by matching the coverage generated by the original reads versus the coverage generated by the binned reads. Bowtie2 [46] was used to map the reads to the reference; parameters: (bowtie2 -f -p 20 -x ChominisCrypto -U original_readsCrypto.fna -S original_cryptomap.sam) and (bowtie2 -f -p 20 -x ChominisCrypto -U binning_readsCrypto.fna -S binning_cryptomap.sam). Figure 5 shows the obtained coverage; the data were plotted on the same figure using another in-house script plot (plotMapping.py original_cryptomap.depth binning_cryptomap.depth).

Additionally we analyzed the biggest bins produced by CLAME (Table 4 and 5). Each bin was assembled using Newbler [14], it was set to minimum identity (mi=95) and minimum length (ml=60) (runAssembly -mi 90 -ml 60 -cpu 8 -o Ensamble_bin12 932332Bin12.fna) (runAssembly -mi 90 -ml 60 -cpu 8 -o Ensamble_bin9 514053Bin9.fna). Annotation, for the Large contigs (>500 bases) was done using AMPHORA2 [17], RAIphy [47] and MEGAN [23]. AMPHORA2 and RAIphy were executed with default parameters (MarkerScanner.pl -DNA Ensamble_bin12/454LargeContigs.fna, MarkerAlignTrim.pl -WithReference -OutputFormat phylip, Phylotyping.pl -CPUs 6 > phylotype.result) (MarkerScanner.pl -DNA Ensamble_bin9/454LargeContigs.fna, MarkerAlignTrim.pl -WithReference -OutputFormat phylip, Phylotyping.pl -CPUs 6 > phylotype.result) and (raiphy -i Ensamble_bin12/454LargeContigs.fna -o raiphyOutput.txt), (raiphy -i Ensamble_bin9/454LargeContigs.fna -o raiphyOutput.txt). For MEGAN, we generated a BLASTn-comparison file of the Large Contigs (>500 bases) against a local NT (downloaded on May 2017) in XML format (blastn -query Ensamble_bin12/454LargeContigs.fna -db /home/db/NT/NT_May_2017/nt -outfmt 5 -num_threads 20 > blastn_rawContigs.xml), (blastn -query Ensamble_bin9/454LargeContigs.fna -db /home/db/NT/NT_May_2017/nt -outfmt 5 -num_threads 20 > blastn_ rawContigs.xml).

San Vicente hot spring metagenome

San Vicente is a hot spring within the Cerro-Machin-Cerro-Bravo volcanic complex in Colombian Andes, located at 4° 50.25' N and 75° 32.35' W at an altitude of 1,715 masl. It is characterized by waters with discharge temperatures above 60 ºC (max. 91 ºC), pH of 6.7 and high concentrations of chlorides. To reduce the complexity of the community, we incubated a sample of the hot spring (discharge temperature 64 ºC) in a non-selective mineral medium, maintained at 45ºC with white light during 15 days (Figure 6). We extracted the community DNA using PowerMax® Soil DNA Isolation Kit supplied by MOBIO Corporation [48], following the instructions of the manufacturer. The sample was sequenced using ROCHE’s 454 Titanium technology in 3/4 PTP at the Centro Nacional de Secuenciación Genómica - CNSG, Universidad de Antioquia, Medellin, Colombia. A total of 926,130 reads (available in CLAME’s GitHub) were generated with a 300bp average length. Raw reads were trimmed using Prinseq [43] tool to keep reads at least 50 bases long, and that at the 3’ the quality is at least 30 (prinseq-lite.pl -fasta termal_reads.fna -out_format 3 -no_qual_header -out_good cleaned_reads -out_bad null -min_len 50 -rm_header -trim_qual_right 30 -trim_qual_type min). Finally, a total of 900,370 quality reads were obtained for further processing steps. The analysis followed in two directions: 1) A de-novo metagenome assembly of the cleaned reads using popular state of the art tools (see below) and further comparison and annotation; 2) the binning of the quality reads using CLAME and further assembly and annotation using the biggest bin.

De-novo assembly was done with Newbler [14], Ray [16] and MetaVelvet [18] (see Table 6). Newbler assembly was set to minimum identity (mi=95) and minimum length (ml=60) (runAssembly -o EnsambleCleanReadsTermal -ml 60 -mi 95 -cpu 8 cleaned_reads.fastq). Ray assembly was configured to use 31 k-mers (mpiexec -n 8 Ray -k 31 -s cleaned_reads.fastq -o ray_Ensamble). MetaVelvet assembly was executed with 31 k-mers (velveth assemblyVelvet 31 -fasta -short cleaned_reads.fna). Annotation, for the Large contigs (>500 bases) reported by Newbler, was done using AMPHORA2 [17], RAIphy [47] and MEGAN [23]. AMPHORA2 was executed with default parameters (MarkerScanner.pl -DNA EnsambleCleanReadsTermal/454LargeContigs.fna, MarkerAlignTrim.pl -WithReference -OutputFormat phylip, Phylotyping.pl -CPUs 6 > phylotype.result). RAIphy was executed with default parameters (raiphy -i EnsambleCleanReadsTermal/454LargeContigs.fna -o raiphyOutput.txt). For MEGAN, we generated a BLASTx-comparison file of the Large contigs (>500 bases) against a local NR in XML format (downloaded on April 2016) (blastx -query EnsambleCleanReadsTermal/454LargeContigs.fna -db /home/db/NR/NR_abril_2016/nr -outfmt 5 -num_threads 20 > blastx_rawContigs.xml). Figure 7 summarizes these results.

Binning process with CLAME was executed using70 bases alignment and without edge threshold restrictions (clame -multiFasta cleaned_reads.fastq -b 70 -output clame_termal -print -fastq). The number of edges histogram is shown in Figure 8 (plotted with the script plotHist.py -f clame_termal.links -c 1 -line -lu 200). Using the Edge analysis stage, CLAME was executed again using 70 bases and restriction for the range 30 edges lower bound and 130 edges upper bound (clame -multiFasta cleaned_reads.fastq -b 70 -e 30,131,10000 -output clame_termal -print -fastq). Only the biggest bin was conserved for further analysis.

Assembly for the biggest bin was done using Newbler [14], Ray [16] and MetaVelvet [18] (see Table 7 and Figure 9). Newbler parameters were: minimum identity 95 and minimum length 60 (runAssembly -o 380846_Newbler -ml 60 -mi 95 -cpu 8 380846fromBiggestBin1.fastq). Ray was configured to use 31 k-mers (mpiexec -n 8 Ray -k 31 -s 380846fromBiggestBin1.fastq -o ray_our_dir). MetaVelvet was executed with 31 k-mers (velveth assemblyVelvet 31 -fasta -short 380846fromBiggestBin1.fna). Large contigs generated by Newbler were classified with AMPHORA2 [17], RAIphy [47], and MEGAN [23] (default parameters as described above) (Figure 10 and 11). For MEGAN, we previously generated a BLASTx-XML comparison file of the Large contigs (>500 bases). The assembly completeness for Newbler’s contigs was measured in terms of gene content and Universal Single-Copy Orthologs presence.

Putative open reading frames (ORFs) were detected using CheckM [49], Prodigal [50] and Genmark [51] tools (Table 8). Parameters: (checkm 380846_Newbler/454LargeContigs.fna checkM_ORFs.faa), (prodigal -i 380846_Newbler/454LargeContigs.fna -o prodigal_ORFs -a prodigal_ORFs.faa), (gmsn.pl 380846_Newbler/454LargeContigs.fna > genMark_ORFs.faa). Quality control for the ORFs reported by Prodigal was done using BLASTp [38] against the NR database from NCBI (blastp -query prodigal_ORFs.faa -db /home/db/NT/NT_abril_2016/nr -outfmt 5 -num_threads 20 > blastp_orfs.xml). Then we employed MEGAN [23] to assign each ORFs into a taxonomic level (Figure 12). Universal Single-Copy Orthologs analysis was done using BUSCO tool [52], parameters (Busco prodigal_ORFs.faa).

Initial taxonomical classification of the organisms represented within the resultant assembled contig set was done searching contigs that contain 16S ribosomal gene sequences. The selected contigs were manually curated, annotated (Table 9) and used to build an evolutionary tree (Figure 13). The phylogenetic tree was inferred by using the Maximum Likelihood method with the Jukes-Cantor model [53] and the process described by Brumm et al. [54]. We conserved the same number of replicates (500) and bootstrapped tree topology to represent the evolutionary history of the taxa analyzed. We used Brumm et al, strategy to obtain the initial tree(s). However, our analysis involved 29 nucleotide sequences, instead of 26 samples. There were a total of 547 positions in the final dataset. All the analysis were developed on MEGA 7.0 [55].

In order to get an insight into the functional annotation of the predicted proteome of the *Xanthomodaceae* of the San Vicente Hot spring, Gene Ontology annotation was performed for the 2,726 ORFs predicted by Prodigal (Figure 14, 15 and 16). It was done using BLASTp comparisons of all the predicted peptides against the NCBI's protein NR database and BLAST2GO version 2.8 [56] annotation tool. Additionally KAAS (KEGG Automatic Annotation Server) [57] was employed to provide a detail functional annotation of predicted genes.

We compared CLAME against MetaProb [28], BiMeta [29], AbundanceBin [31] and MetaBinG [27] tools. The parameters used were (BiMeta -finput cleaned_reads.fna -numSp 5 -l 30 -m 15 -ssize 20 -read 0), (MetaProb -si cleaned_reads.fna -numSp 5 -feature 2 -m 45), (abundancebin -input cleaned_reads.fna -output cleaned_readsBin -bin_num 5), ((CPU version) metabing cleaned_reads.fna > cleaned_readsBin.txt). For the tools in which the number of bins or species can be specified, we decided to set it to 5, according the number of phyla found by the annotation tools described previously. The biggest bins reported by each tool were assembled using Newbler [14], it was setting at minimum identity (mi=95) and minimum length (ml=60) in all the cases. Table 10 compares these results versus CLAME’s de-novo assembly for the biggest bin.

We also analyzed the other bins (with at least 2,000 reads) produced by CLAME. These bins were assembled with Newbler [14], minimum identity (mi=95) and minimum length (ml=60), and annotated with MEGAN [23], AMPHORA2 [17] and RAIphy [47]. AMPHORA2 and RAIphy were executed with default parameters and for MEGAN we generated a BLASTn-comparison file of the Large contigs (>500 bases) against a local NT (downloaded on May 2017) in XML format.

In order to study the other species presents in the metagenome, we elaborated an auxiliary dataset by deleting the reads binned in the first CLAME execution and conserved the balance of the read in the original dataset. A total of the 519,524 reads conform this second dataset. CLAME was executed on this dataset using 15 bases matching and edge thresholds for the range 10 to 20 (Figure 17), only bins with at least 2,000 reads were reported (clame -b 15 -e 10,21,10000 -multiFasta balance_of_380846.fna -output clame_balance -print -sizeBin 2000). The parameters were configured experimentally to get suitable bins. The biggest bin produced by CLAME was assembled with Newbler [14] and annotated using Megan [23], AMPHORA2 [17] and RAIphy [47] (Table 11 and 12). AMPHORA2 and RAIphy were executed with default parameters. For MEGAN we generated a BLASTn-comparison file of the Large contigs (>500 bases) against a local NT (downloaded on May 2017) in XML format.

CLAME computational performance

We show CLAME’s speed and memory performances on Figure 18 and 19. All the experiments were performed on a computer equipped with 64 Intel(R) Xeon(R) CPU X7560 @ 2.27GHz and 500 GB of RAM. CLAME was implemented in C ++ using OpenMP (Open Multi-Processing) interface. We executed CLAME employing 1, 2, 4, 8, 16, 32 and 64 threads on each dataset previously explained. We selected the best of five executions. Valgrind [58] was used to measure CLAME’s memory usage. We took the maximal memory usage of each experiment.
