# Supplementary material for: CLAME: a new alignment-based binning algorithm allows the genomic description of a novel Xanthomonadaceae from the Colombian Andes
Source: BMC Genomics. 2018 Dec 11;19(Suppl 8):858. doi: 10.1186/s12864-018-5191-y (PMC6288851; doi:10.1186/s12864-018-5191-y)
Supplement: Supplementary file 2 — Full table list . (DOCX 26 kb) [file 12864_2018_5191_MOESM2_ESM.docx]

**CLAME: Supplementary-Material document**

**Tables**

Table 1. Bins reported by each tool on the simulated metagenome. It also shows the number of reads that belong to each genome for each bin, and the time it took each tool to create the bins.

| Tool | Bins | Total reads by bin | B. Cannis | M. Tuberculosis | Time  (m) |
| --- | --- | --- | --- | --- | --- |
| CLAME | 2 | 353876 | **0** | 353876 | 8 |
|  |  | 280014 | 280014 | **0** |  |
| BiMeta | 2 | 8990 | 8683 | 307 | 49 |
|  |  | 656049 | 366439 | 289610 |  |
| MetaProb | 2 | 368642 | 2901 | 365787 | 12 |
|  |  | 296397 | 287062 | 9335 |  |
| AbundanceBin | 2 | 659892 | 288233 | 371659 | 85 |
|  |  | 5142 | 1684 | 3458 |  |
| MetaBinG | 2 | 600615 | 5215 | 295400 | 97 |
|  |  | 338650 | 267794 | 70856 |  |

Table 2. Species and total reads used to create the simulated multi-species metagenome. It shows the size of the original database, in reads and bases, the reads and bases used to create the metagenome, the size of the reported genome, and the depth calculated as the bases used divided by the genome size.

| Species | NCBI reference | Phylum/Class | Total  reads | Total  bases  (Mbp) | Used  reads | Used  bases  (Mpb) | Genome  size  (Mpb) | Depth(x) |
| --- | --- | --- | --- | --- | --- | --- | --- | --- |
| Synecho-cystis | DRR106442 | Cyanobacteria/Cyanobacteria | 589689 | 21.9 | 112805 | 41.5 | 3.5 | 11.7 |
| Dokdo-nella | SRR4217676 | Proteobacteria/Gammaproteo-bacteria | 376022 | 80.5 | 376022 | 80.5 | 4.6 | 17.41 |
| Hymno-bacter | SRR1334914 | Bacteroidetes/ Cytophagia | 2917298 | 958.5 | 37599 | 12.3 | 5.0 | 2.4 |
| Micro-bacteriaceae | SRR5493999 | Actinobacteria/Actinobacteria | 1815433 | 382.4 | 37599 | 7.9 | 3.2 | 2.4 |
| Rhizo-bium | SRR5165471 | Proteobacteria/Alphaproteo-bacteria | 1152754 | 242.2 | 37599 | 7.9 | 4.5 | 1.7 |

Table 3. Bins reported by the binning tools on the simulated multi-species metagenome. It also shows the number of reads that belong to each genome for each bin, and the time it took each tool to create the bins.

| Tool | Bins | Total reads by bin | Synecho-cystis | Dokdo-nella | Hymno-bacter | Micro-bacteriaceae | Rhizo-bium | Time (m) |
| --- | --- | --- | --- | --- | --- | --- | --- | --- |
| CLAME | 7 | 21182 | 21182 | **0** | **0** | **0** | **0** | 3 |
|  |  | 18054 | 18054 | **0** | **0** | **0** | **0** |  |
|  |  | 209642 | **0** | 209642 | **0** | **0** | **0** |  |
|  |  | 12152 | **0** | 12152 | **0** | **0** | **0** |  |
|  |  | 13927 | **0** | 13927 | **0** | **0** | **0** |  |
|  |  | 10405 | **0** | 10405 | **0** | **0** | **0** |  |
|  |  | 24315 | **0** | **0** | **0** | 24315 | **0** |  |
| BiMeta | 1 | 601624 | 112805 | 376022 | 37599 | 37599 | 37599 | 32 |
| MetaProb | 5 | 361966 | 1 | 341866 | 108 | 7236 | 12755 | 11 |
|  |  | 27977 | 508 | 12139 | 1707 | 214 | 13409 |  |
|  |  | 113349 | 111889 | 695 | 641 | 6 | 118 |  |
|  |  | 38400 | 294 | 729 | 34383 | 2446 | 548 |  |
|  |  | 59932 | 113 | 20593 | 760 | 27697 | 10769 |  |
| MetaBinG | 5 | 410033 | 30727 | 302805 | 23480 | 19944 | 33081 | 120 |
|  |  | 73263 | 799 | 57637 | 3915 | 9490 | 1423 |  |
|  |  | 61401 | 56764 | 2344 | 772 | 1211 | 310 |  |
|  |  | 24966 | 18955 | 3042 | 1079 | 870 | 1021 |  |
|  |  | 10826 | 12 | 3800 | 6444 | 436 | 134 |  |

Table 4. Assembly statistics of the biggest bins reported by CLAME on the Illumina metagenome.

| Bin number | Total reads | Large contigs | Expected genome size (Mbp) | AVG contig length (bp) | Largest contig (bp) | N50 | GC (%) |
| --- | --- | --- | --- | --- | --- | --- | --- |
| 12 | 932332 | 3211 | 6.0 | 1867 | 60200 | 2639 | 37.67 |
| 9 | 514053 | 447 | 3.6 | 8112 | 85325 | 22568 | 56.58 |

Table 5. Annotation of Newbler’s Large contigs assembled from the biggest bins reported by CLAME on the Illumina metagenome.

| Contigs | MEGAN | | RAIphy | | AMPHORA2 | |
| --- | --- | --- | --- | --- | --- | --- |
|  | Total Contigs/  Phylum | Total Contigs/  Species | Total Contigs/  Phylum | Total Contigs/  Species | Total Contigs/  Phylum | Total Contigs/  Species |
| 3211 from the bin 12 | 2856/ Firmicutes | 2409/ Veillonella | 2896/ Firmicutes | 2437/ Veillonella | 39/ Firmicutes | 38/ Veillonella |
| 447 from the bin 9 | 301/  Actino-bacteria | 300 /  Bifido-bacterium | 259 /  Actino-bacteria | 237 /  Bifido-bacterium | 40 /  Actino-bacteria | 39/  Bifido-bacterium |

Table 6. Assembler statistic reported by each tool on the original hot spring dataset, without binning.

|  | Total large contigs (>500bp) | Reads assembled | Largest contig (bp) | Expected genome size (Mbp) | N50 | AVG contig length (bp) | Peak depth | GC (%) |
| --- | --- | --- | --- | --- | --- | --- | --- | --- |
| Newbler | 11739 | 804983 (87%) | 232982 | 27 | 3267 | 2349 | 2.1 | 61 |
| Ray | 12369 | 768803 (83%) | 72115 | 14 | 1143 | 1134 | 4.8 | 61 |
| MetaVelvet | 17720 | 797792 (86%) | 7084 | 19 | 1199 | 1104 | 2.6 | 61 |

Table 7. Assembler statistic reported by each tool on the hot spring dataset of the biggest bin produced by CLAME.

|  | Total large contigs (>500bp) | Reads assembled | Largest contig (bp) | Expected genome size (Mbp) | N50 | AVG contig length (bp) | Peak depth | GC (%) |
| --- | --- | --- | --- | --- | --- | --- | --- | --- |
| Newbler | 178 | 380796 (99%) | 99748 | 3.0 | 31130 | 17067 | 60 | 71 |
| Ray | 255 | 372145 (97%) | 72110 | 3.0 | 19598 | 20242 | 23 | 71 |
| MetaVelvet | 712 | 371284 (97%) | 26703 | 2.9 | 6816 | 4135 | 40 | 71 |

Table 8. Gene composition analysis for the Newbler’s Large contigs assembled of CLAME’s biggest bin of the hot spring metagenome.

|  | CheckM | Prodigal | Genmark |
| --- | --- | --- | --- |
| Total ORFs | 2726 | 2726 | 2661 |
| Number of contigs | 173 | 173 | 168 |
| ORFs distribution | 0.96 | 0.96 | 0.86 |

Table 9. BLASTn top 7 hits report for the 16S rRNA gene sequence found in the Newbler’s contig00154 of the assembly of CLAME largest bin of the hot spring metagenome.

|  | Score (Bits) | Ident (%) | E-Value | Accession |
| --- | --- | --- | --- | --- |
| Uncultured bacterium clone 16S-27F&1492R-C12-clone6 | 2241 | 99 | 0.0 | KX348539.1 |
| Uncultured bacterium clone B63 | 2228 | 99 | 0.0 | AF407725.1 |
| Uncultured bacterium clone EG90 | 2044 | 95 | 0.0 | KC189660.1 |
| Uncultured bacterium clone JN11 | 2039 | 95 | 0.0 | JN868991.1 |
| Uncultured bacterium clone LONG_SPR_11F | 2026 | 95 | 0.0 | KF836265.1 |
| *Metallibacterium scheffleri* strain DKE6 | 1891 | 93 | 0.0 | NR_118103.1 |
| *Dokdonella koreensis* DS-123 | 1874 | 92 | 0.0 | CP015249.1 |

Table 10. Newbler assembly statistics of the bins reported by each tools on the hot spring metagenome. It also shows the time it took each tool to create the bins.

| Tool | Total Bins | Total reads | Large contigs | Expected genome size (Mbp) | AVG contig length (bp) | Largest contig (bp) | N50 | GC (%) | Time (m) |
| --- | --- | --- | --- | --- | --- | --- | --- | --- | --- |
| CLAME | 2 | 380846 | 178 | 3.03 | 17067 | 99748 | 31130 | 71 | 9 |
|  |  | 446 | 24 | 25157 | 1048 | 2791 | 1054 | 66.17 |  |
| BiMeta | 5 | 113070 | 2131 | 2.3 | 1082 | 28701 | 1077 | 65 | 211 |
|  |  | 22877 | 728 | 0.6 | 867 | 6907 | 860 | 38 |  |
|  |  | 273565 | 995 | 2.98 | 3002 | 49922 | 11620 | 72 |  |
|  |  | 283509 | 3499 | 5.95 | 1701 | 45994 | 2185 | 70 |  |
|  |  | 207349 | 3857 | 9.73 | 2523 | 41372 | 4961 | 51 |  |
| MetaProb | 5 | 275160 | 3423 | 5 | 1460 | 53631 | 1561 | 69 | 21 |
|  |  | 60580 | 1350 | 1.3 | 966 | 11767 | 966 | 58 |  |
|  |  | 204718 | 4262 | 9.45 | 2217 | 29837 | 4059 | 51 |  |
|  |  | 47618 | 766 | 0.7 | 901 | 6858 | 898 | 61 |  |
|  |  | 312294 | 1486 | 4.7 | 3149 | 63982 | 6146 | 72 |  |
| AbundanceBin | 3 | 459353 | 950 | 3.7 | 3876 | 75296 | 12564 | 69 | 1063 |
|  |  | 190112 | 6574 | 8.1 | 1240 | 8964 | 1475 | 56 |  |
|  |  | 250905 | 8938 | 8.6 | 968 | 4762 | 1005 | 62 |  |
| MetaBinG | 3 | 521865 | 7765 | 9.8 | 1253 | 30729 | 1278 | 66 | 131 |
|  |  | 212100 | 3115 | 4.6 | 1480 | 9988 | 1829 | 71 |  |
|  |  | 125979 | 4764 | 6.7 | 1400 | 13502 | 1647 | 51 |  |

Table 11. Thermal metagenome Newbler assembler statistics for the balance reads (without the reads used for the draft genome).

| Bin number | Total of reads | Total large contigs | Largest contig (bp) | Expected genome size (Mbp) | N50 | AVG contig length (bp) | GC (%) |
| --- | --- | --- | --- | --- | --- | --- | --- |
| 0 | 146977 | 5056 | 8852 | 5.9Mpb | 1277 | 1163 | 51.58 |

Table 12. Annotation of Newbler’s Large contigs assembled from the thermal metagenome from the balance reads (without the reads used for the draft genome).

| Phylum | MEGAN | RAIphy | AMPHORA 2 |
| --- | --- | --- | --- |
| Cyanobacteria | 3214 (63.57 %) | 3339 (66.04 %) | 37 (0.73 %) |
| Proteobacteria | 167 (3.30 %) | 1161 (22.96 %) | 2 (0.04 %) |
| Bacteroidetes | 18 (0.36 %) | 36 (0.71 %) | 2 (0.04 %) |
| Others | 411 (8.13 %) | 520 (10.28 %) | 1 (0. 019 %) |
| Unknown | 1246 (24.64 %) | 0 (0.00 %) | 5014 (99.17 %) |
