# Supplementary material for: CLAME: a new alignment-based binning algorithm allows the genomic description of a novel Xanthomonadaceae from the Colombian Andes
Source: BMC Genomics. 2018 Dec 11;19(Suppl 8):858. doi: 10.1186/s12864-018-5191-y (PMC6288851; doi:10.1186/s12864-018-5191-y)
Supplement: Supplementary file 3 — Full figures file. (PDF 2788 kb) [file 12864_2018_5191_MOESM3_ESM.pdf]

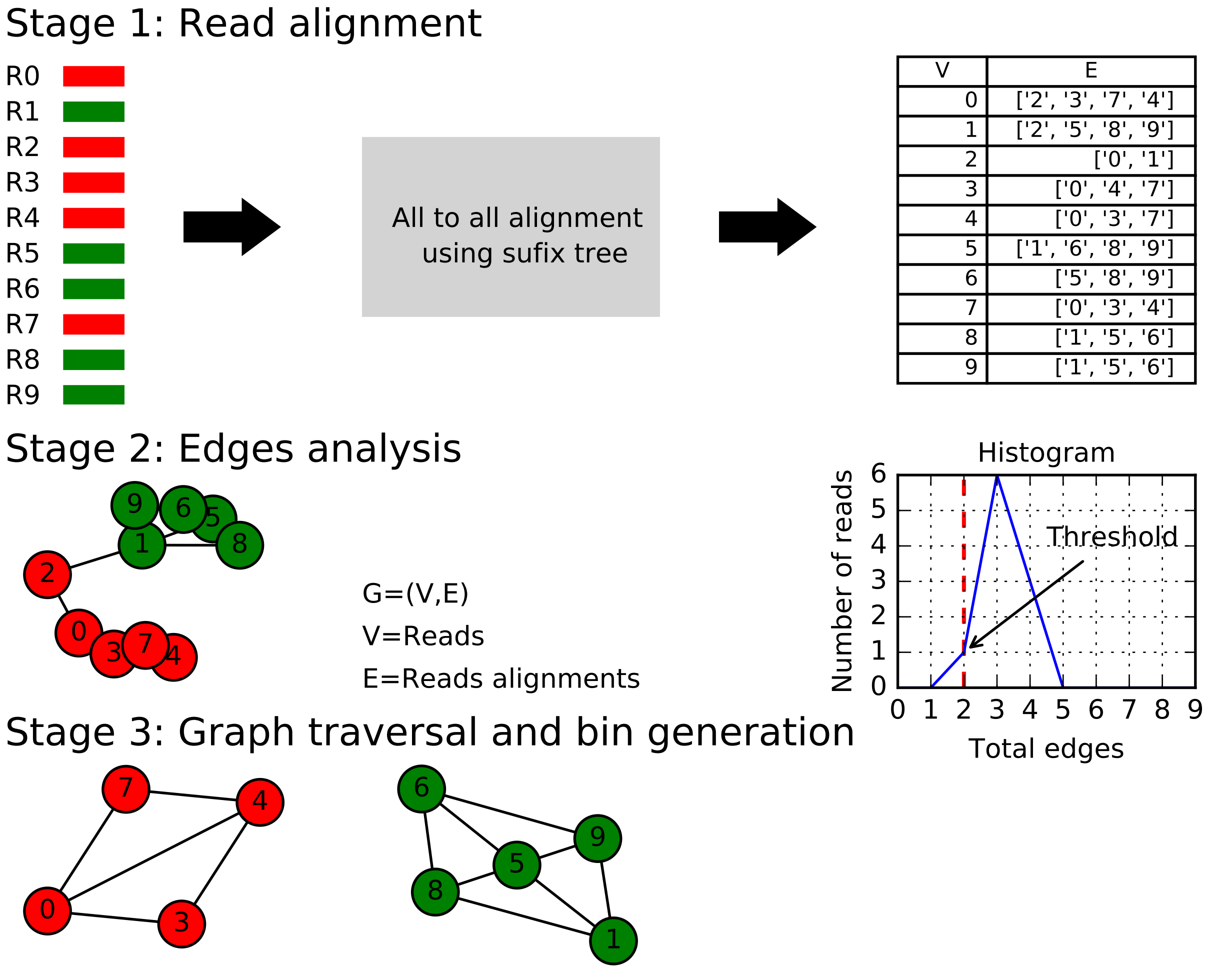

V	E
0	['2', '3', '7', '4']
1	['2', '5', '8', '9']
2	['0', '1']
3	['0', '4', '7']
4	['0', '3', '7']
5	['1', '6', '8', '9']
6	['5', '8', '9']
7	['0', '3', '4']
8	['1', '5', '6']
9	['1', '5', '6']

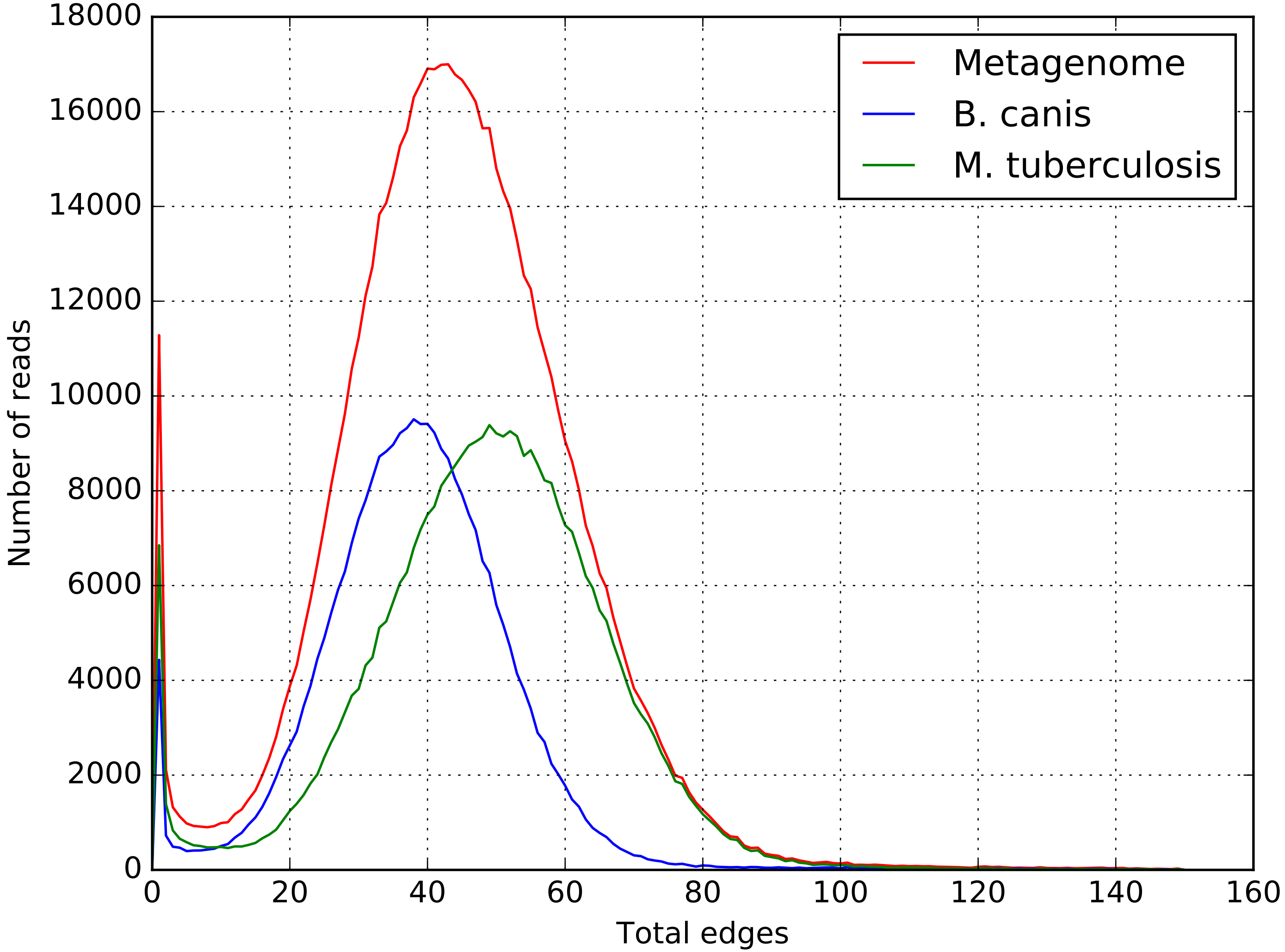

Metagenome
B. canis
M. tuberculosis
Number of reads
Total edges
0
2000
4000
6000
8000
10000
12000
14000
16000
18000
0
20
40
60
80
100
120
140
160

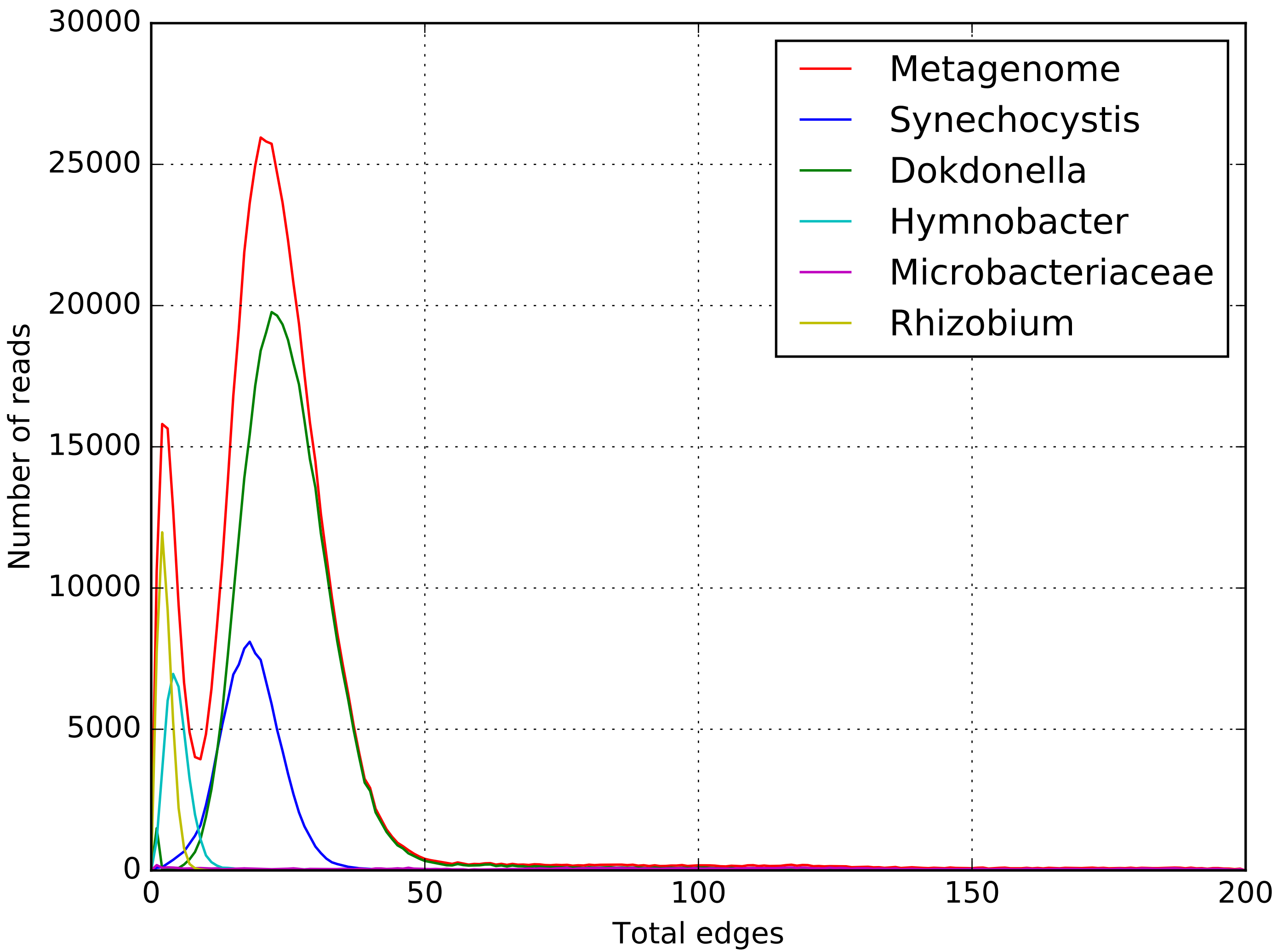
Metagenome
Synechocystis
Dokdonella
Hymnobacter
Microbacteriaceae
Rhizobium
Number of reads
Total edges
0
5000
10000
15000
20000
25000
30000
0
50
100
150
200

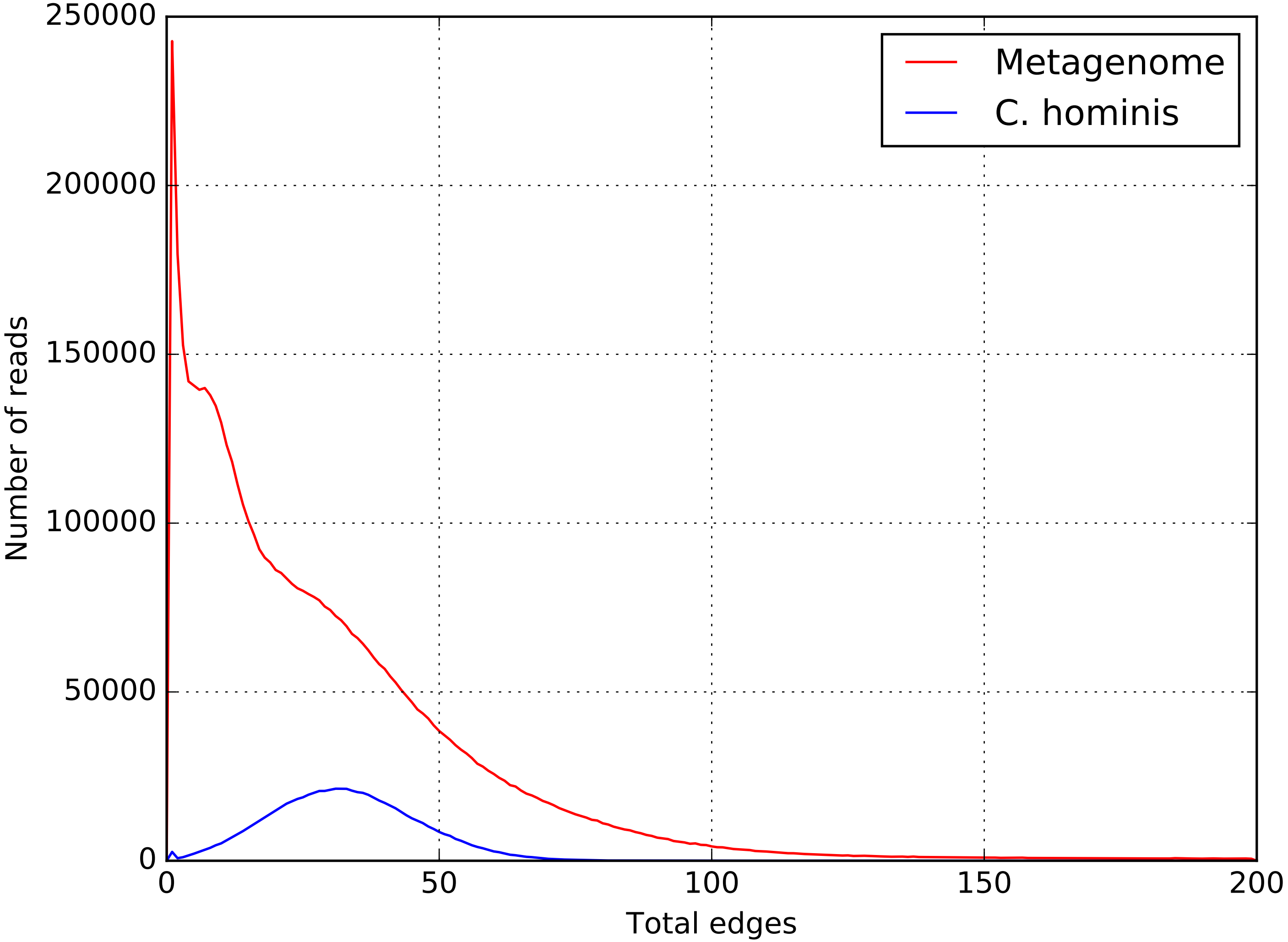

250000
200000
150000
100000
50000
0
0
50
100
150
200
Total edges
Number of reads
Metagenome
C. hominis

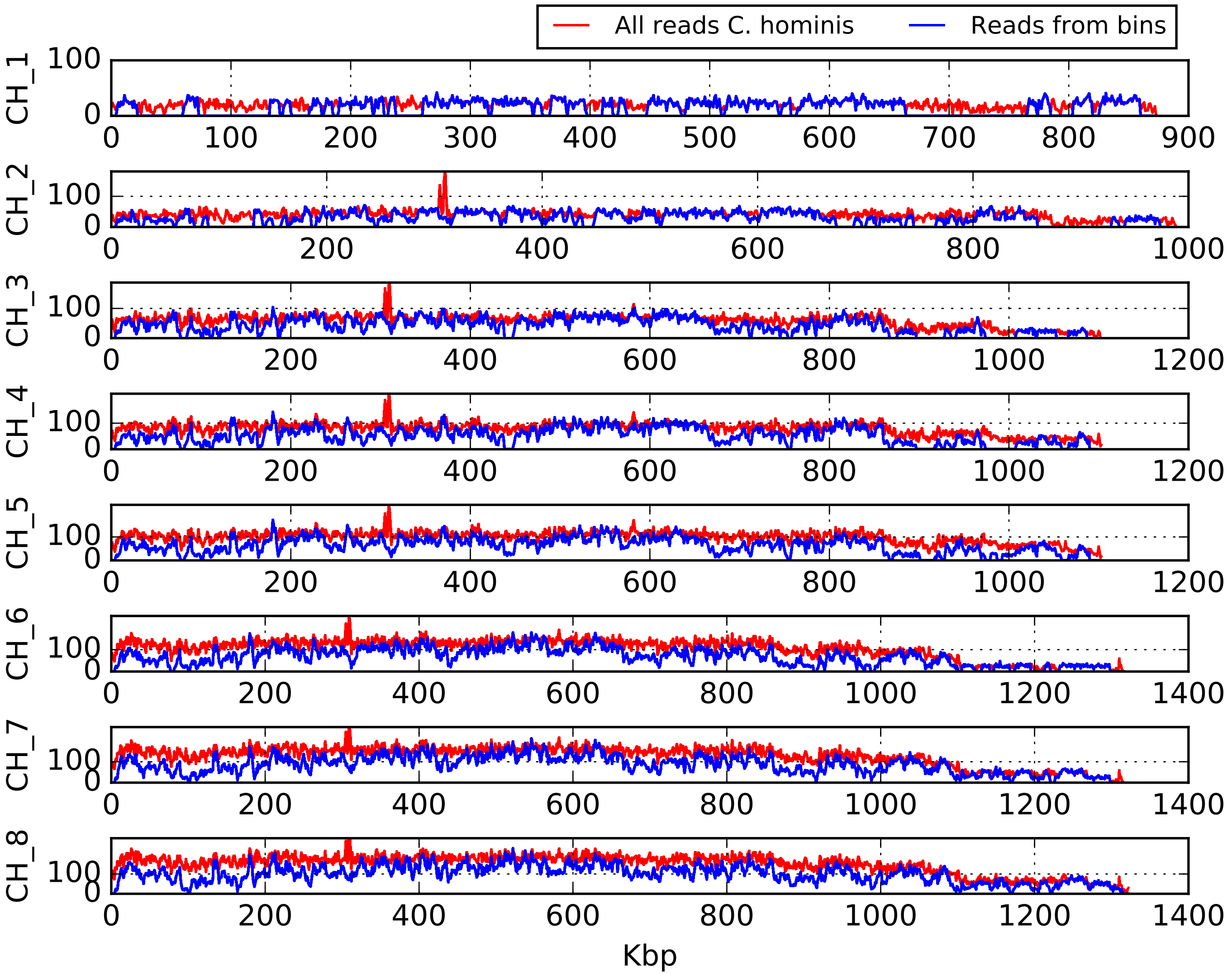

All reads C. hominis
Reads from bins
CH_1
CH_2
CH_3
CH_4
CH_5
CH_6
CH_7
CH_8
0
100
200
300
400
500
600
700
800
900
1000
1200
1400
Kbp

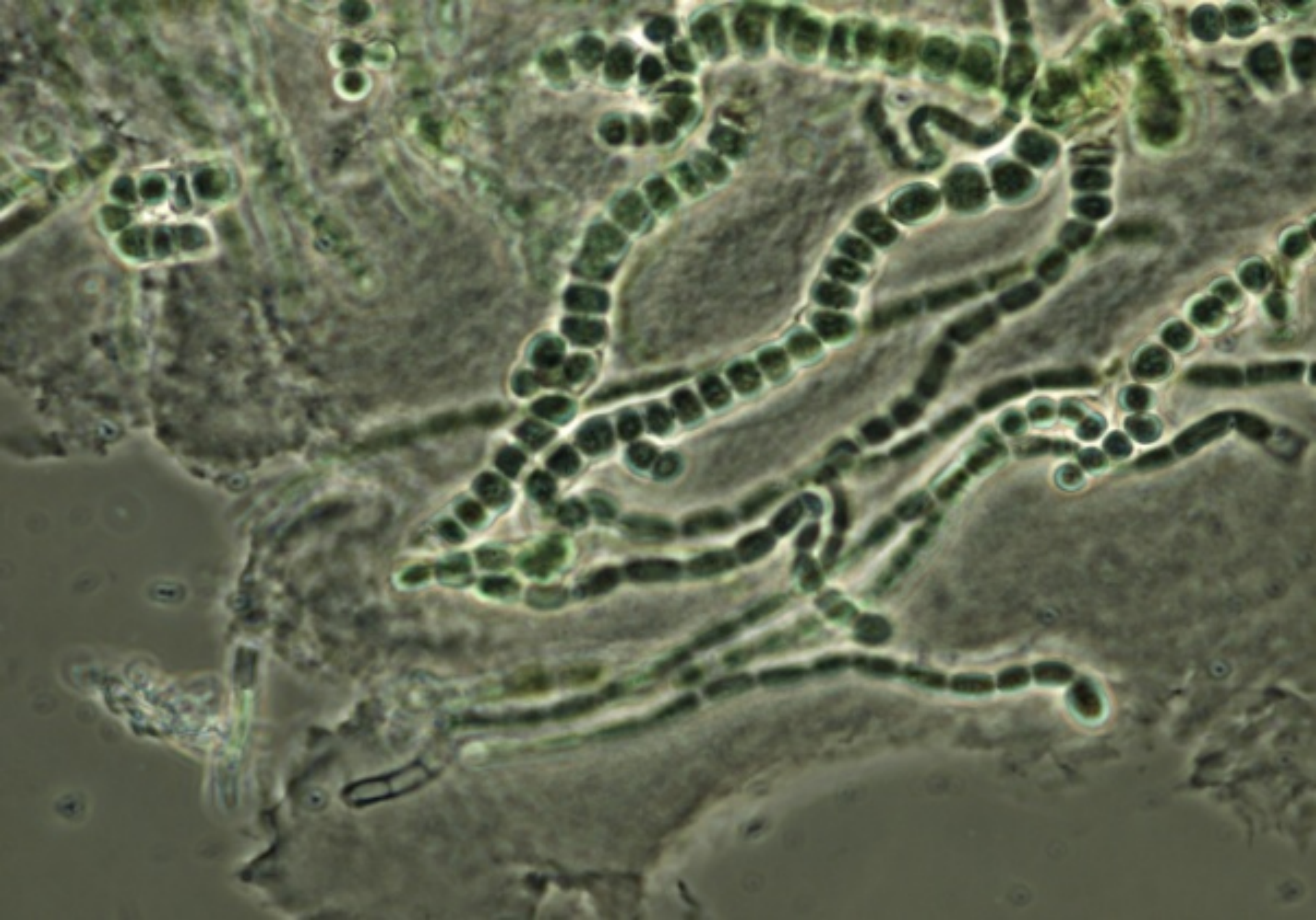

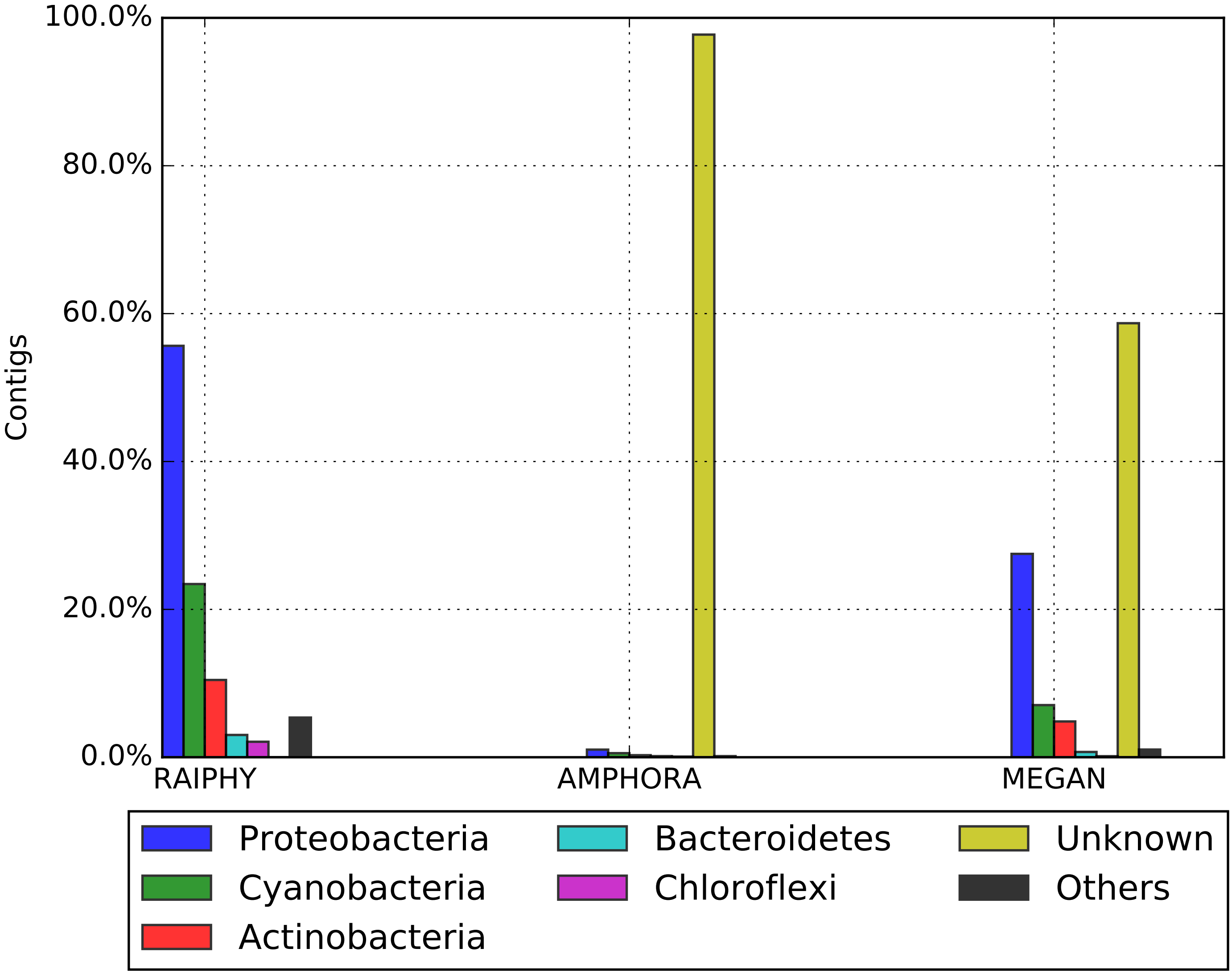

100.0%
80.0%
60.0%
40.0%
20.0%
0.0%
Contigs
RAIPHY
AMPHORA
MEGAN
Proteobacteria
Cyanobacteria
Actinobacteria
Bacteroidetes
Chloroflexi
Unknown
Others

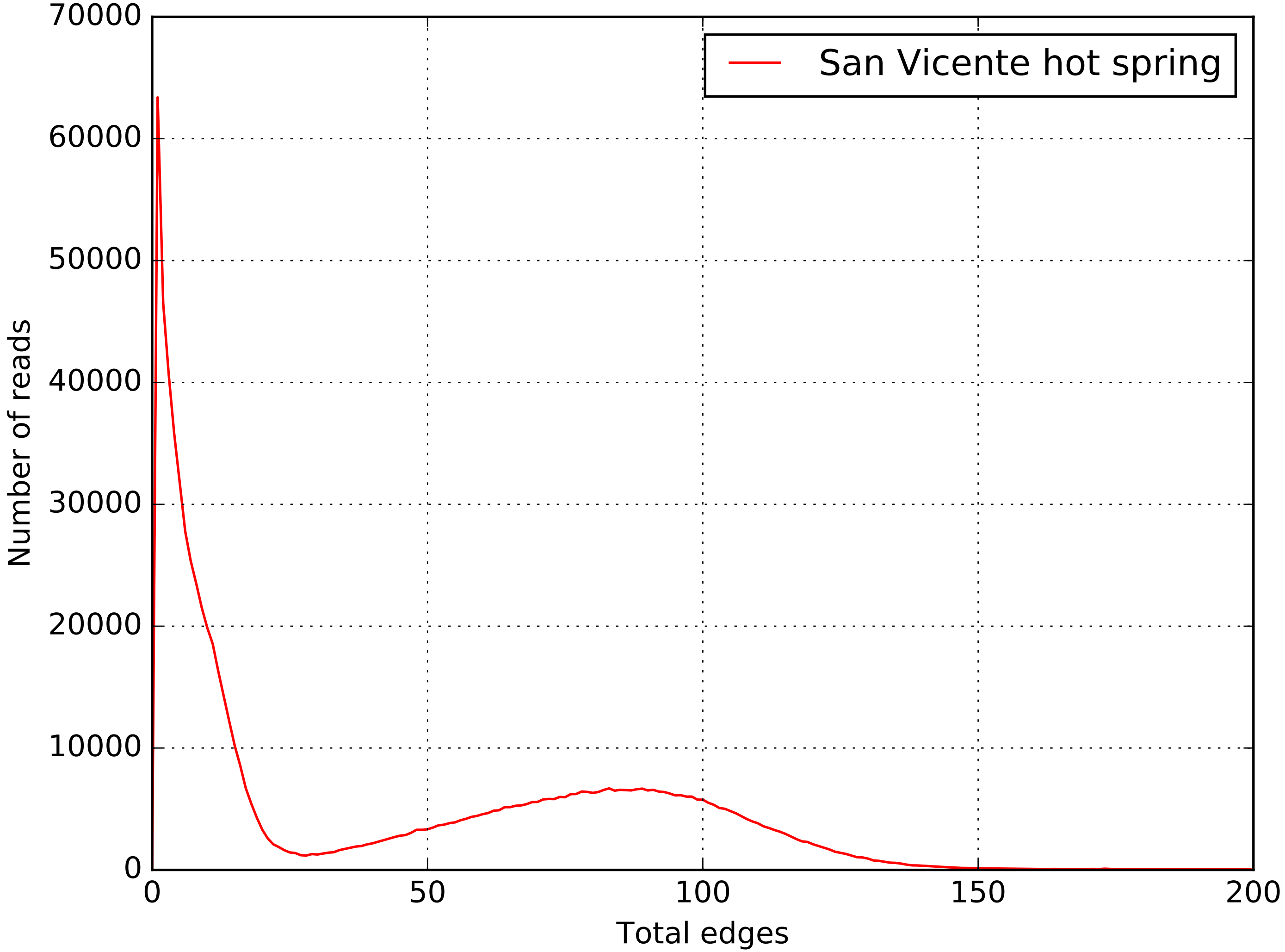

San Vicente hot spring
Number of reads
Total edges
0
10000
20000
30000
40000
50000
60000
70000
0
50
100
150
200

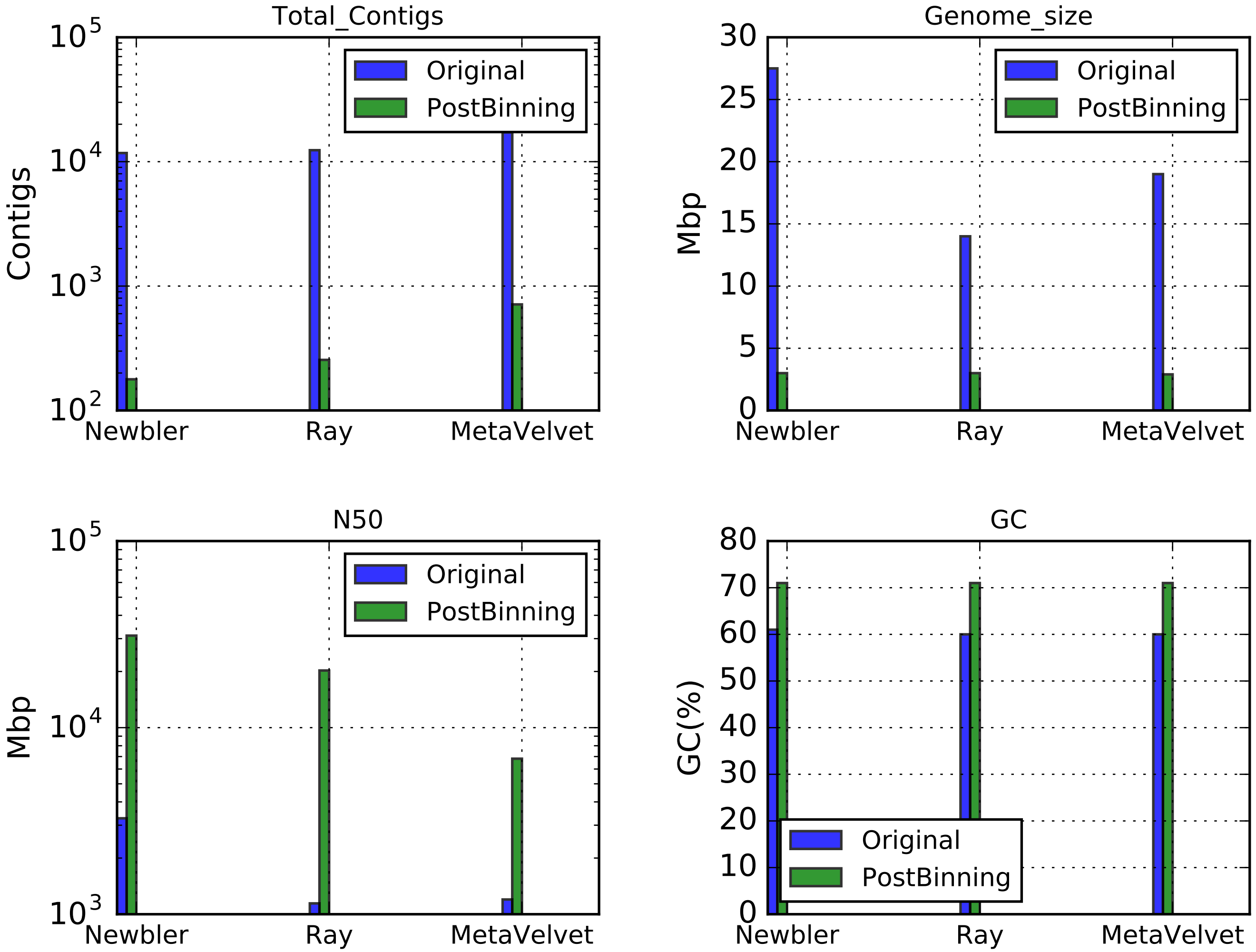
Total_Contigs
Contigs
Original
PostBinning
Newbler
Ray
MetaVelvet
Genome_size
Mbp
N50
GC
GC(%)

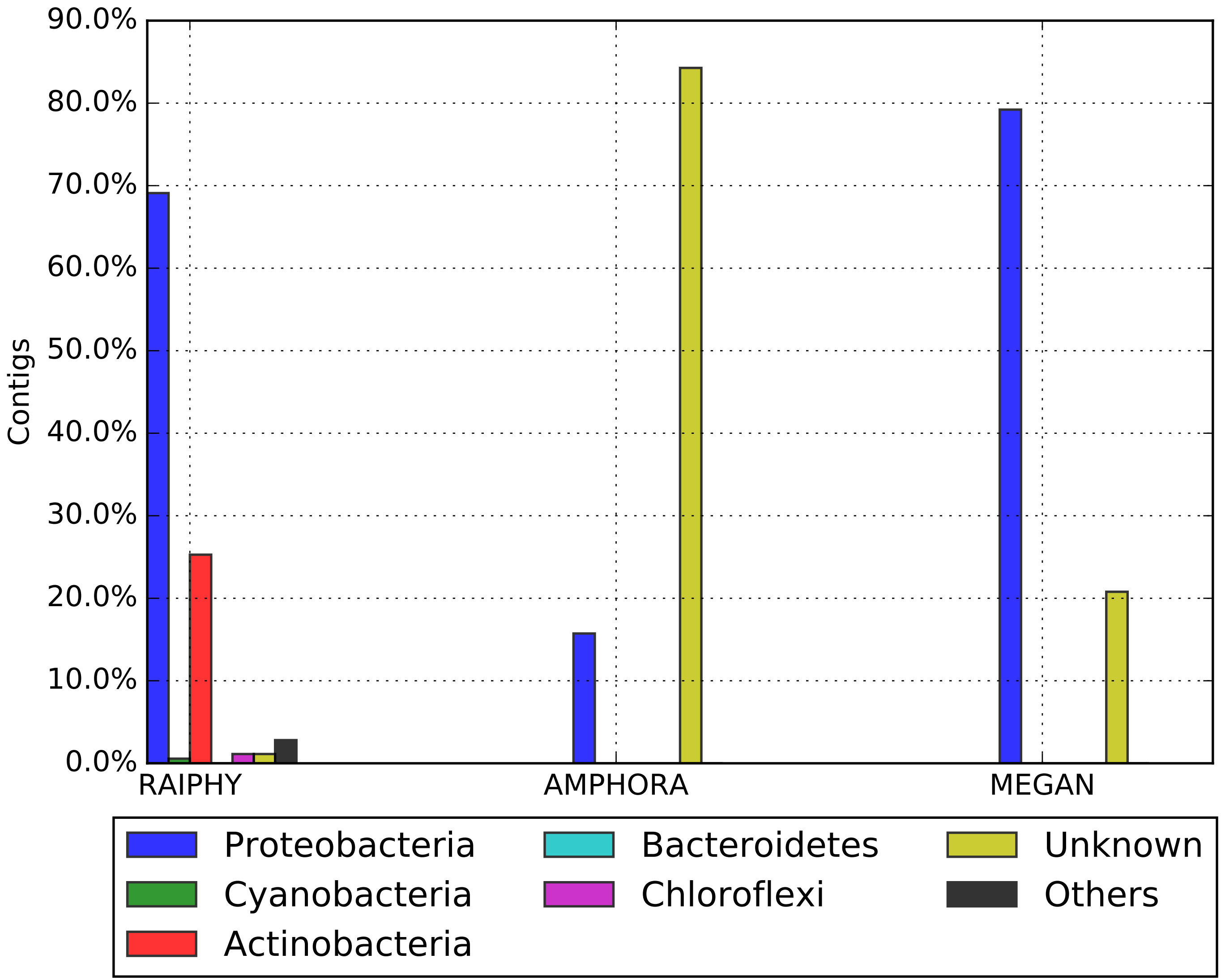

90.0%
80.0%
70.0%
60.0%
50.0%
40.0%
30.0%
20.0%
10.0%
0.0%
Contigs
RAIPHY
AMPHORA
MEGAN
Proteobacteria
Cyanobacteria
Actinobacteria
Bacteroidetes
Chloroflexi
Unknown
Others

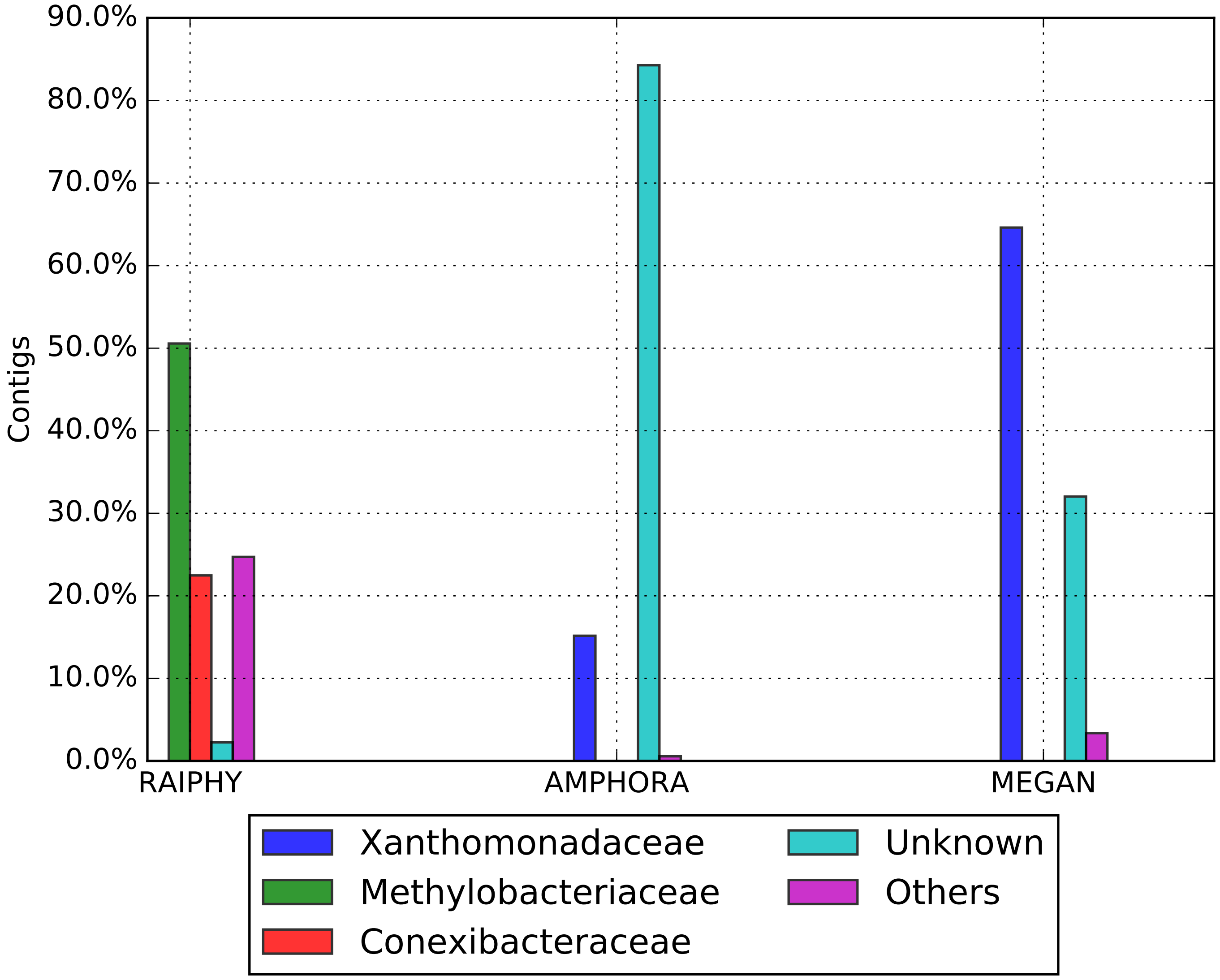
90.0%
80.0%
70.0%
60.0%
50.0%
40.0%
30.0%
20.0%
10.0%
0.0%
Contigs
RAIPHY
AMPHORA
MEGAN
Xanthomonadaceae
Methylobacteriaceae
Conexibacteraceae
Unknown
Others

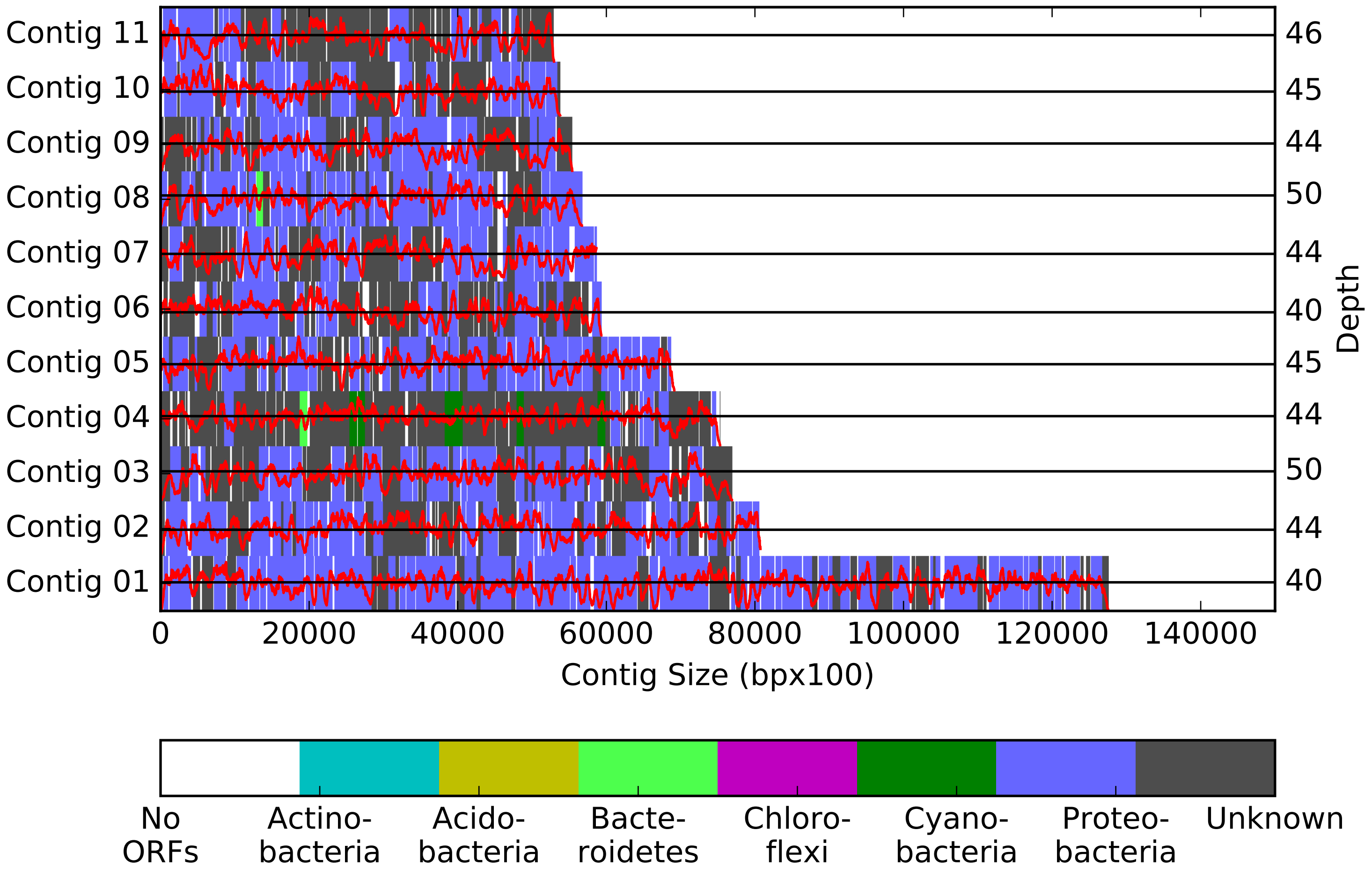

Contig 11
Contig 10
Contig 09
Contig 08
Contig 07
Contig 06
Contig 05
Contig 04
Contig 03
Contig 02
Contig 01
46
45
44
50
44
40
45
44
50
44
40
Depth
0
20000
40000
60000
80000
100000
120000
140000
Contig Size (bpx100)
No ORFs
Actino-bacteria
Acido-bacteria
Bacte-roidetes
Chloro-flexi
Cyano-bacteria
Proteo-bacteria
Unknown

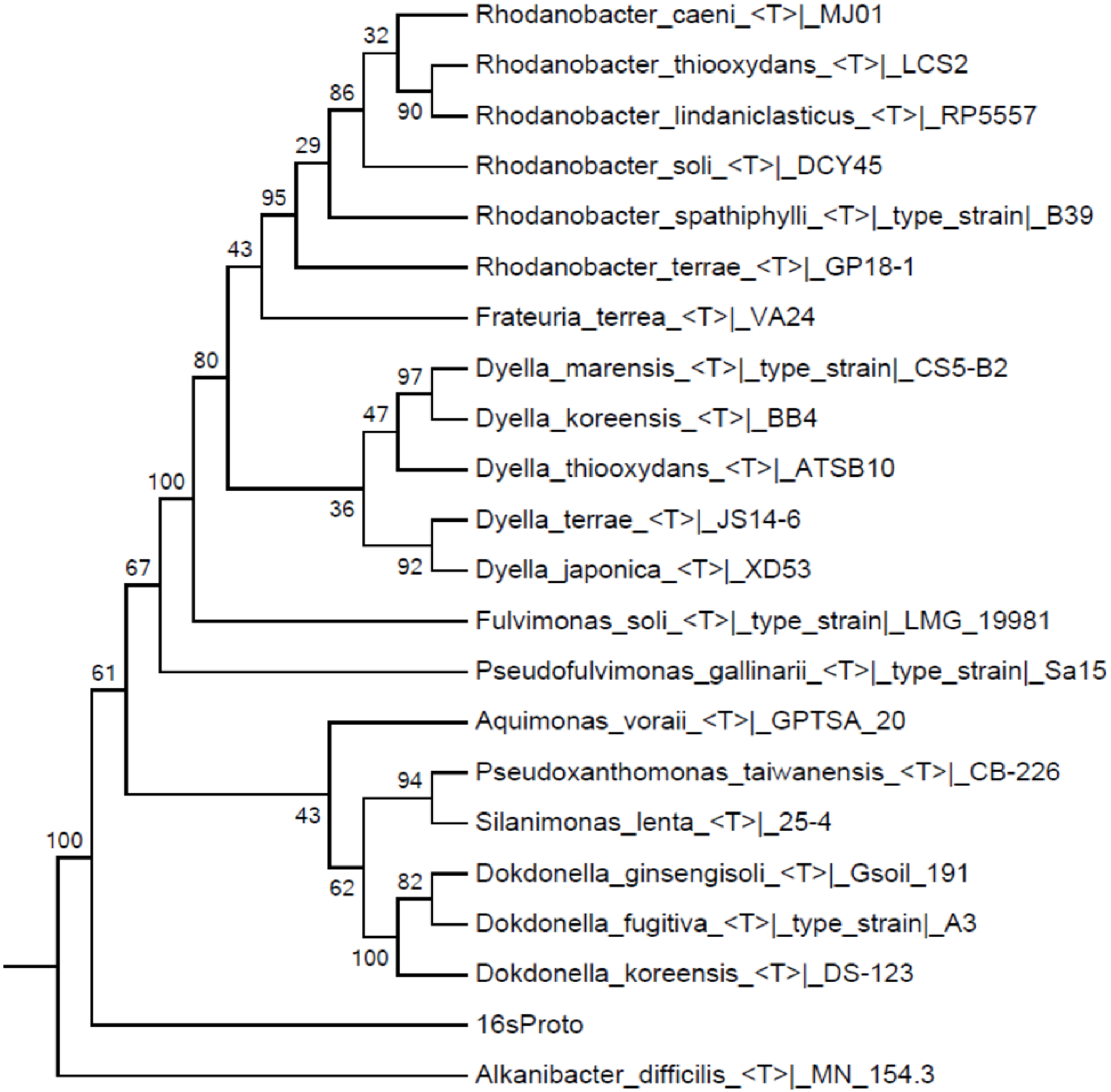

Rhodanobacter_caeni_<T>|_MJ01
Rhodanobacter_thiooxydans_<T>|_LCS2
Rhodanobacter_lindaniclasticus_<T>|_RP5557
Rhodanobacter_soli_<T>|_DCY45
Rhodanobacter_spathiphylli_<T>|_type_strain|_B39
Rhodanobacter_terrae_<T>|_GP18-1
Frateuria_terrea_<T>|_VA24
Dyella_marensis_<T>|_type_strain|_CS5-B2
Dyella_koreensis_<T>|_BB4
Dyella_thiooxydans_<T>|_ATSB10
Dyella_terrae_<T>|_JS14-6
Dyella_japonica_<T>|_XD53
Fulvimonas_soli_<T>|_type_strain|_LMG_19981
Pseudofulvimonas_gallinarii_<T>|_type_strain|_Sa15
Aquimonas_voraii_<T>|_GPTSA_20
Pseudoxanthomonas_taiwanensis_<T>|_CB-226
Silanimonas_lenta_<T>|_25-4
Dokdonella_ginsengisoli_<T>|_Gsoil_191
Dokdonella_fugitiva_<T>|_type_strain|_A3
Dokdonella_koreensis_<T>|_DS-123
16sProto
Alkanibacter_difficilis_<T>|_MN_154.3
32
90
86
29
95
43
80
97
47
36
92
100
67
61
43
94
62
82
100
100

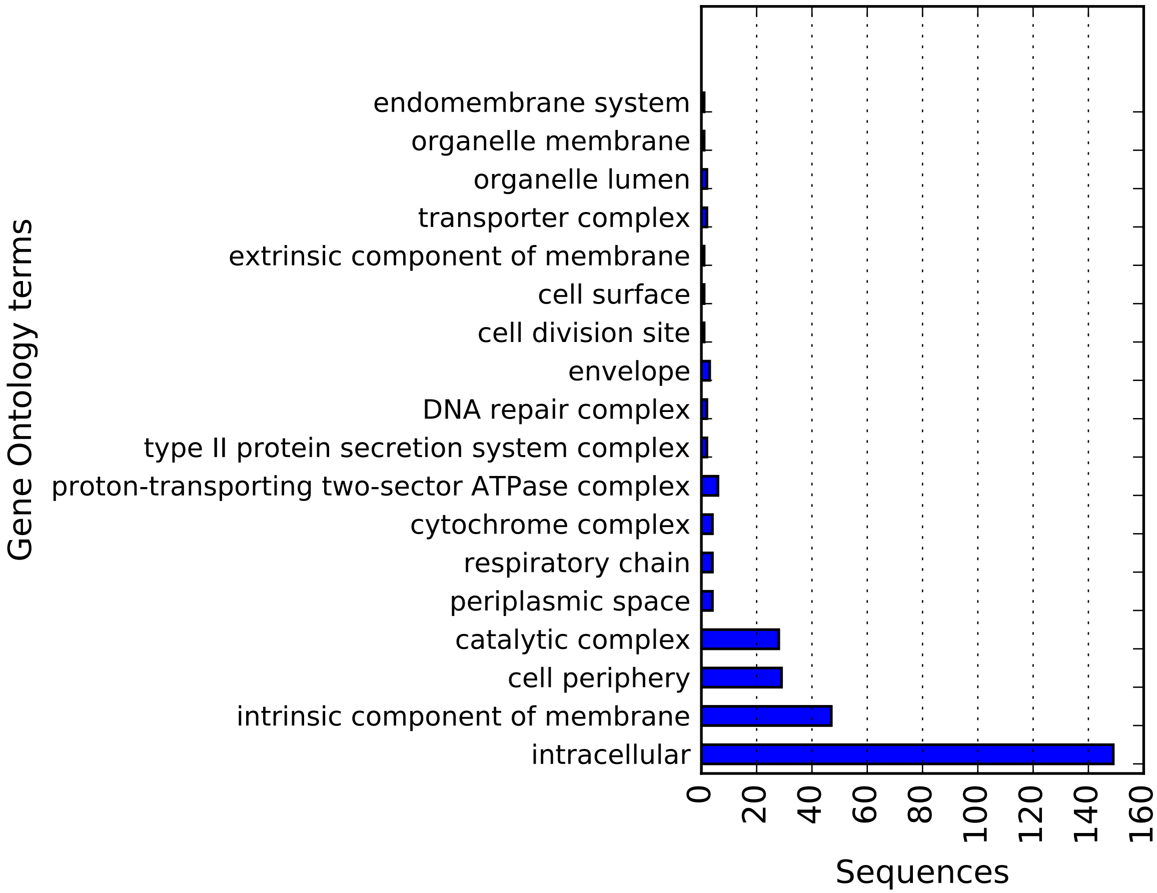
Gene Ontology terms
endomembrane system
organelle membrane
organelle lumen
transporter complex
extrinsic component of membrane
cell surface
cell division site
envelope
DNA repair complex
type II protein secretion system complex
proton-transporting two-sector ATPase complex
cytochrome complex
respiratory chain
periplasmic space
catalytic complex
cell periphery
intrinsic component of membrane
intracellular
0
20
40
60
80
100
120
140
160
Sequences

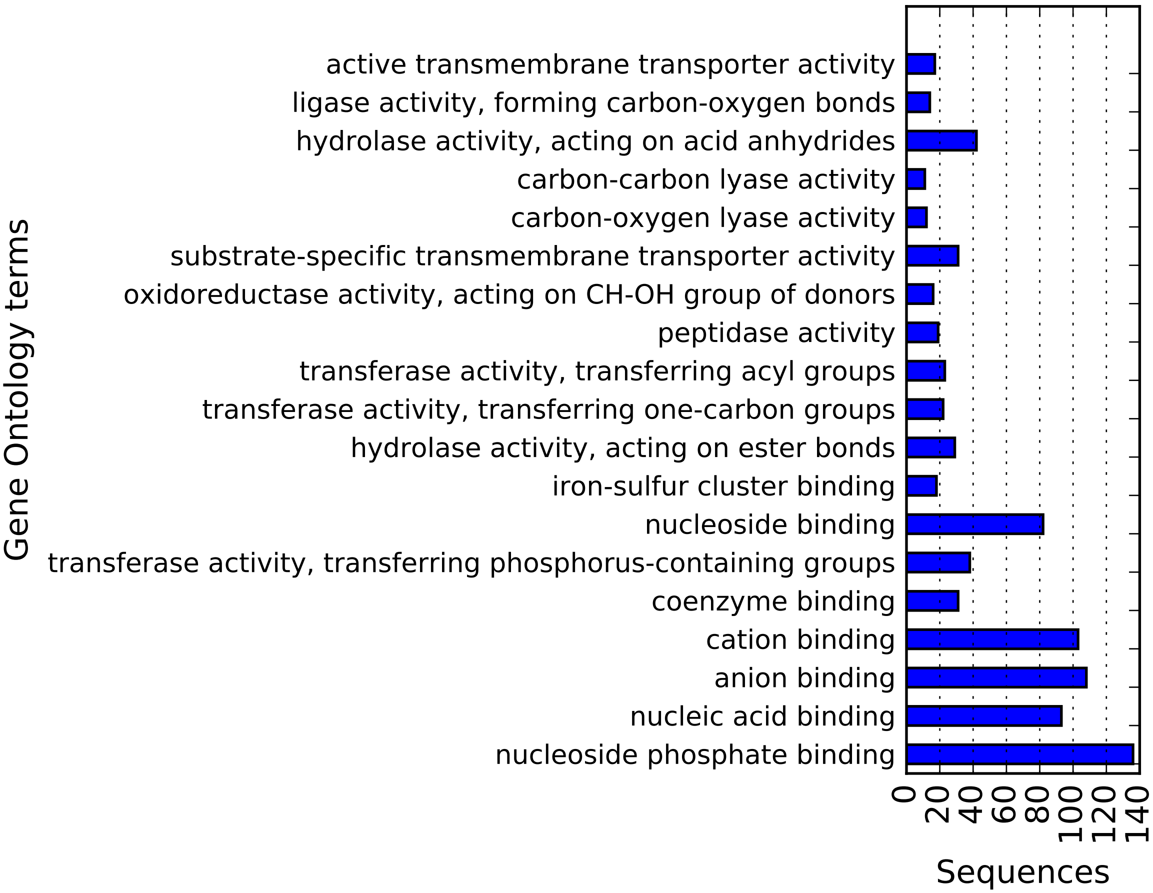
Gene Ontology terms
active transmembrane transporter activity
ligase activity, forming carbon-oxygen bonds
hydrolase activity, acting on acid anhydrides
carbon-carbon lyase activity
carbon-oxygen lyase activity
substrate-specific transmembrane transporter activity
oxidoreductase activity, acting on CH-OH group of donors
peptidase activity
transferase activity, transferring acyl groups
transferase activity, transferring one-carbon groups
hydrolase activity, acting on ester bonds
iron-sulfur cluster binding
nucleoside binding
transferase activity, transferring phosphorus-containing groups
coenzyme binding
cation binding
anion binding
nucleic acid binding
nucleoside phosphate binding
0
20
40
60
80
100
120
140
Sequences

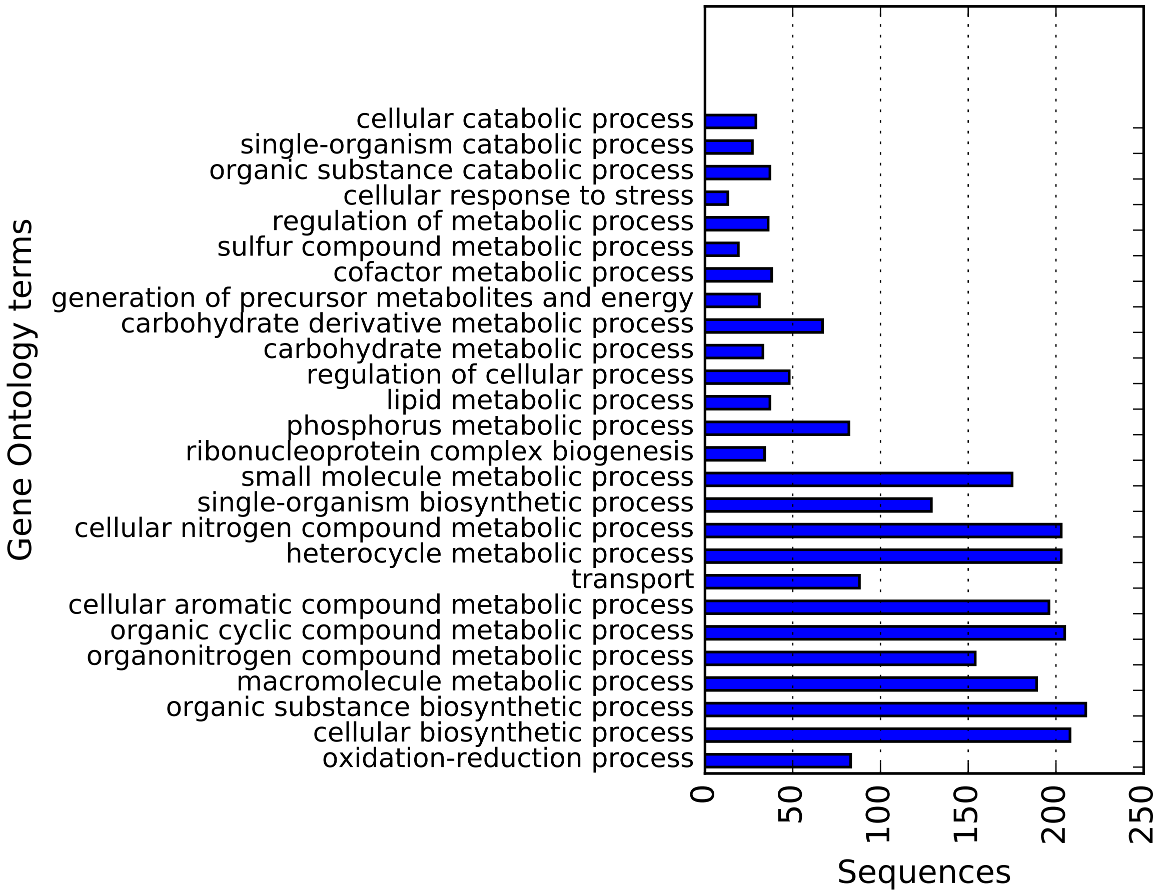
Gene Ontology terms
cellular catabolic process
single-organism catabolic process
organic substance catabolic process
cellular response to stress
regulation of metabolic process
sulfur compound metabolic process
cofactor metabolic process
generation of precursor metabolites and energy
carbohydrate derivative metabolic process
carbohydrate metabolic process
regulation of cellular process
lipid metabolic process
phosphorus metabolic process
ribonucleoprotein complex biogenesis
small molecule metabolic process
single-organism biosynthetic process
cellular nitrogen compound metabolic process
heterocycle metabolic process
transport
cellular aromatic compound metabolic process
organic cyclic compound metabolic process
organonitrogen compound metabolic process
macromolecule metabolic process
organic substance biosynthetic process
cellular biosynthetic process
oxidation-reduction process
0
50
100
150
200
250
Sequences

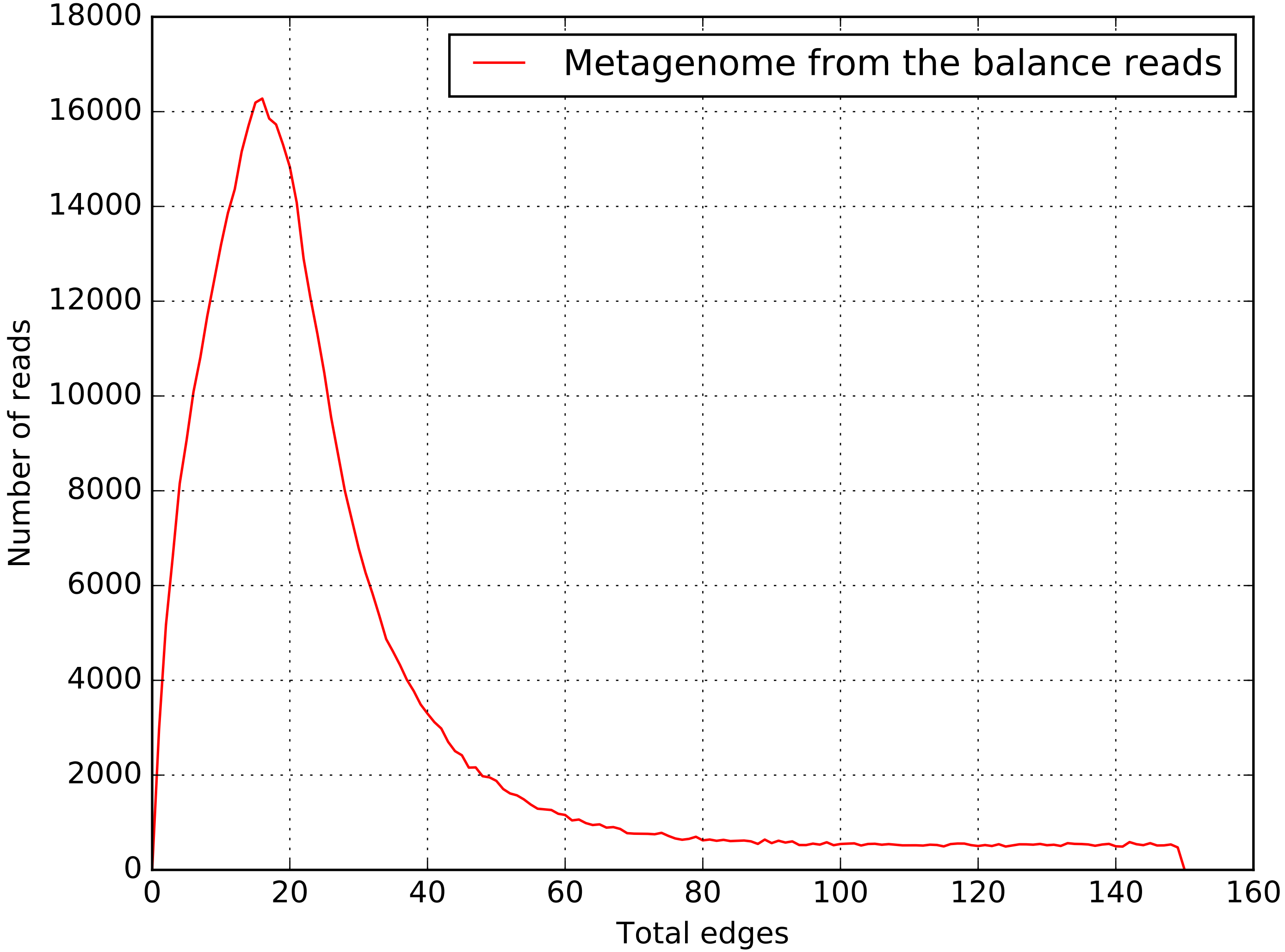

Metagenome from the balance reads
Number of reads
Total edges
18000
16000
14000
12000
10000
8000
6000
4000
2000
0
0
20
40
60
80
100
120
140
160

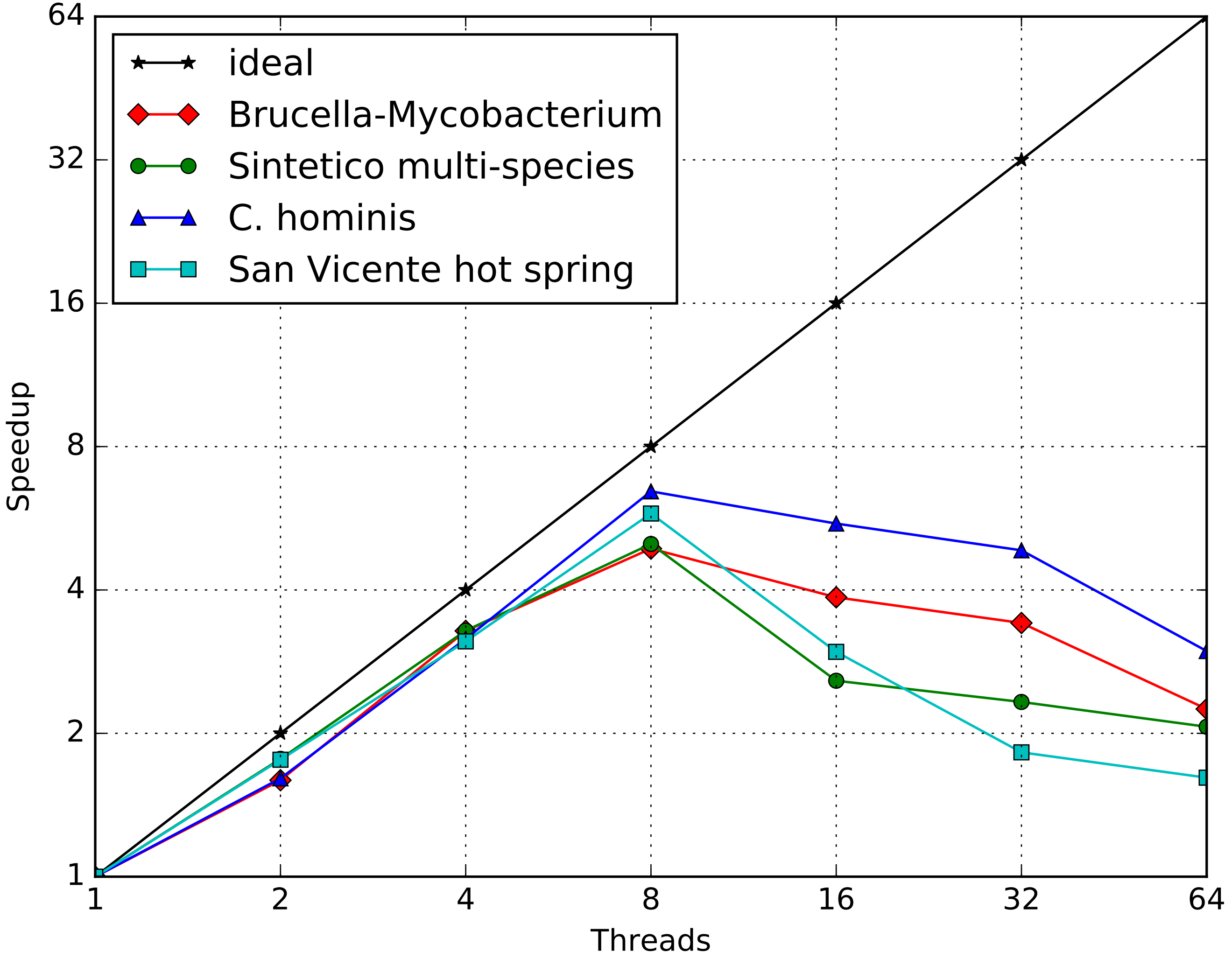
ideal
Brucella-Mycobacterium
Sintetico multi-species
C. hominis
San Vicente hot spring
Speedup
Threads
64
32
16
8
4
2
1
1
2
4
8
16
32
64

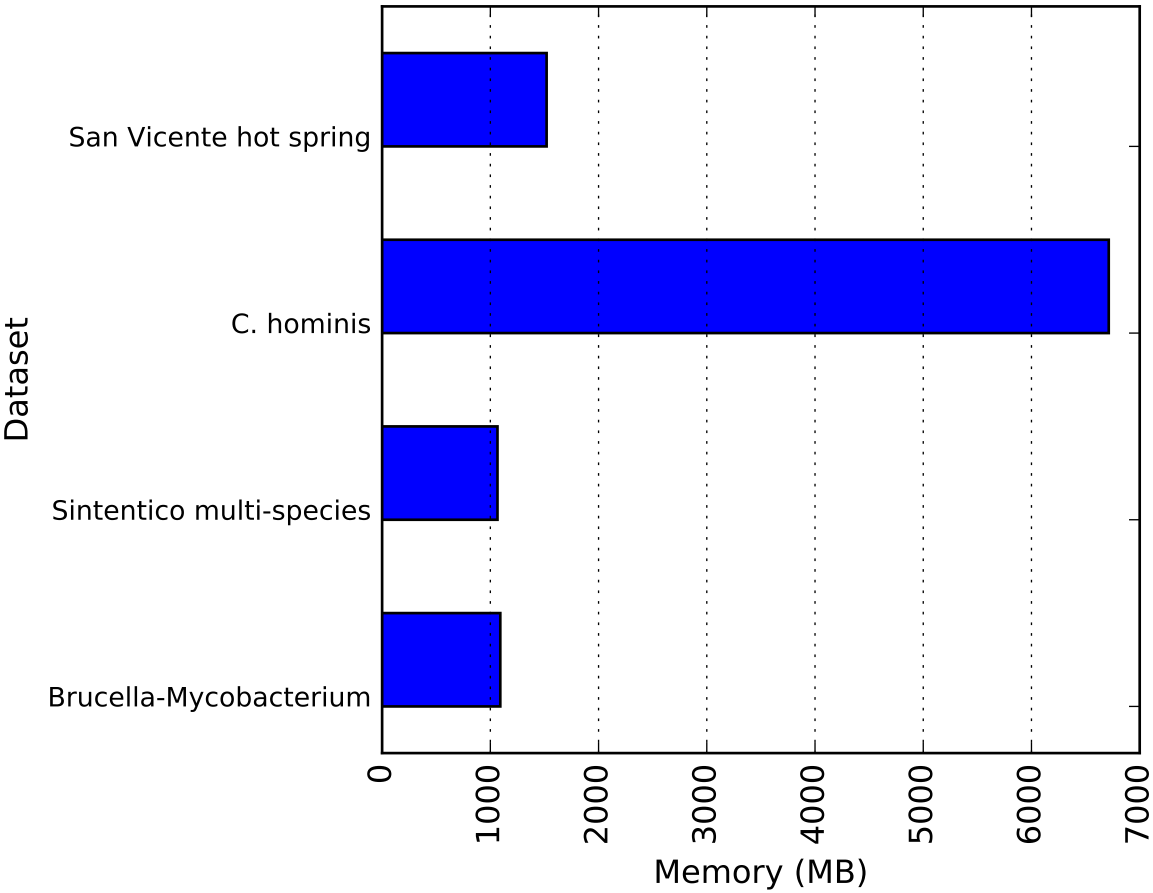

San Vicente hot spring
C. hominis
Sintentico multi-species
Brucella-Mycobacterium
Dataset
0
1000
2000
3000
4000
5000
6000
7000
Memory (MB)
